# A data-driven crop model for biomass sorghum growth process simulation

**DOI:** 10.3389/fpls.2025.1617775

**Published:** 2025-11-13

**Authors:** Yanbin Chang, Zheng Ni, Juan S. Panelo, Joshua Kemp, Maria G. Salas-Fernandez, Lizhi Wang

**Affiliations:** 1 School of Industrial Engineering and Management, Oklahoma State University, Stillwater, OK, United States; 2 Department of Agronomy, Iowa State University, Ames, IA, United States; 3 Horticultural Sciences Department, University of Florida, Gainesville, FL, United States; 4 Department of Bioengineering, George Mason University, Fairfax, VA, United States; 5 Department of Systems Engineering and Operations Research, George Mason University, Fairfax, VA, United States

**Keywords:** biomass sorghum, yield prediction, data-driven crop model, process-based crop model, integrated crop model

## Abstract

Accurate simulation of crop growth processes for predicting final yield is critical for optimizing resource management, particularly in regions with variable climates and limited resource availability. This paper proposes a novel data-driven crop model to simulate phenotypic changes during biomass sorghum growth. The model integrates a detailed physiological framework for sorghum development—tracking how phenotypes are determined by genotype, environment, management practices, and their interactions—with data-driven techniques to calibrate genotypic parameters using experimental data. Results demonstrate that the model achieves accurate biomass production predictions and successfully disentangles the effects of environmental and management factors on phenotypic development, even with limited data. This model enhances the accuracy and applicability of biomass sorghum growth and yield prediction models, offering valuable insights for precision agriculture.

## 1 Introduction

Sorghum (*Sorghum bicolor* (L.) Moench) is a versatile C4 drought-resistant and nutritionally valuable crop, integral to food security and biofuel production around the world (Wang et al., 2008; Xiong et al., 2019; Silva et al., 2022). Among different sorghum types, biomass sorghum has emerged as a resource capable of accumulating over 20 tn/ha of dry matter (Salas-Fernandez and Kemp, 2022) for forage and bioenergy production. In addition, bioenergy sorghums are beneficial toward greenhouse gas mitigation (Olson et al., 2012).

Biomass yield can be influenced by environmental factors, management practices (Olson et al., 2013) and, given its polygenic nature, genotypic variability (Breitzman et al., 2019; Habyarimana et al., 2020; Singh et al., 2025). Key biomass-related traits such as stem diameter, plant height (Salas Fernandez et al., 2017), flowering time (Habyarimana et al., 2020), and carbon partitioning (Boatwright et al., 2022) have been the focus of attention to dissect the complexity of biomass yield. The study of biomass-related traits led to the development of genetic resources including the Bioenergy Association Panel, the Carbon-Partitioning Nested Association Mapping panel and the Photoperiod Sensitive Panel (PSP) (Brenton et al., 2016; Yu et al., 2016; Boatwright et al., 2021). These populations enabled studying biomass-related traits with germplasm relevant to the production system, integrating growth dynamics with high throughput phenotyping (Panelo et al., 2024) and crop modeling strategies (Panelo et al., 2025).

Accurate simulation of the biomass sorghum growth process is pivotal for predicting the final yield and optimizing resource management strategies, particularly in areas susceptible to climate variability and resource constraints (Zha et al., 2010; Biazin et al., 2012; Kugedera et al., 2022). Reliable yield predictions are essential for optimizing agronomic interventions, resource allocation, and supply chain logistics. Consequently, researchers have explored various modeling approaches, ranging from process-based crop simulations to data-driven models, to address this challenge.

Process-based crop models have been widely used to predict sorghum yield by explicitly integrating various physiological processes, environmental factors, and management practices. SORKAM, introduced by Rosenthal et al. (1989), broke ground by modeling daily canopy development and adjusting carbon partitioning based on organs’ demands. This sink-source foundation was brought into the Decision Support System for Agrotechnology Transfer (DSSAT) framework, introducing CERES-Sorghum (Virmani et al., 1989). In CERES-Sorghum, radiation-use efficiency drives daily biomass production that is then distributed to leaves, stems, and grain according to stage-specific coefficients, whereas genotypic coefficients drive mostly crop phenology (White et al., 2015). Continuous updates in the CERES-Sorghum model improved routines for leaf area development and biomass partitioning, boosting predictive skill by up to 20% (White et al., 2015), while experiments with larger rooting depths have successfully identified management practices for sweet sorghum (Lopez et al., 2017). The Agricultural Production Systems sIMulator (APSIM) is another radiation use efficiency-based model, including a sorghum module that has been optimized for integration with plant breeding (Hammer et al., 2010). This crop growth model has been effective for simulating genetic diversity in sorghum across environments (MacCarthy et al., 2009; Chimonyo et al., 2016; Truong et al., 2017; Yang et al., 2021; Tirfessa et al., 2023). Advancements in high-throughput phenotyping allowed integrating remotely sensed leaf area index (LAI) and vegetation indices like NDVI with both CERES and APSIM to correct state variables, thus improving predictive ability under varying climatic conditions (Masjedi et al., 2018; Della Nave et al., 2022; Kivi et al., 2023). Generally, process-based models like APSIM and DSSAT describe processes on a fine-scale temporal basis (Jones et al., 2003; Holzworth et al., 2014). However, calibrating the parameters used by process-based models represents a challenge as it requires resource-intensive field experiments in a range of environments (He et al., 2017).

Data-driven models aim to build a mathematical relationship between the input data and the output, unlike process-based models which rely on known physiological mechanisms (Roberts et al., 2017). Jiang et al. (2004) developed an artificial neural network using back-propagation algorithms to enhance crop yield prediction accuracy. Over a decade later, several deep neural network based models were developed to ingest daily weather grids, layered soils, and genotype markers to untangle genotype by environment (G×E) interactions driving yield. Khaki and Wang (2019) predicted maize yields for new hybrids planted in unseen locations by learning the complex G×E interactions from historical trials, while Shook et al. (2021) integrated genotype information with weather variables to improve soybean yield prediction. Later, Khaki et al. (2020) improved generalization by introducing convolutional neural networks and recurrent neural networks (CNN-RNN) framework which extracts spatio-temporal features from weather and soil data to capture latent G×E patterns. Such hybrid CNN-RNN outperformed random forests and linear models (Khaki et al., 2020).

Statistical regression modeling is another data-driven method, which can take advantage of weather and remote sensing data. County-scale weather regressions could achieve notable accuracy in maize yield forecasting (Conradt et al., 2016), satellite-derived vegetation indices, weather, soil, and location data could explain the soybean yield variation (Chen et al., 2019). Similar methods using NDVI time-series have been applied for wheat yield estimation as well (Duan et al., 2017). These studies highlight that well-structured regression models can provide robust, interpretable predictions, especially when paired with remote sensing and meteorological inputs. Likewise with process-based models, integration of high-throughput phenotyping imagery from unmanned aerial vehicles (UAVs) and advanced machine learning improve precision. Varela et al. (2021) demonstrated that high temporal resolution UAV imagery can capture growth dynamics in biomass sorghum by extracting time-series features as canopy development rates. Their model utilized dynamic and time-point specific image-derived features to predict biomass accumulation, highlighting the benefit of monitoring crop progress over time. Integration of UAV-based data with deep learning algorithms sharped predictive performance, as the fine-scale, high-resolution data from UAVs better capture crop health and stress status throughout the growing season (Masjedi et al., 2019; Khaki et al., 2021; Wang and Crawford, 2021; Wang et al., 2023). The data-driven modeling approach has two major limitations. First, the black-box structure between input and output layers makes the results less interpretable since it can build relationships in the data that do not consider known assumptions (Alibabaei et al., 2022; Drees et al., 2024). Second, the model performance is highly sensitive to data quantity and quality, posing challenges when applying the model with insufficient or noisy data (Jabed and Murad, 2024; Miftahushudur et al., 2025).

Researchers have recently attempted to integrate traditional process-based crop growth models with data-driven modeling techniques to gain both accuracy and interpretability. One popular route treats simulated state the variables as engineered features, fed into gradient-boosting or bagged-tree ensembles (Feng et al., 2020; Shahhosseini et al., 2021). They demonstrated how output variables from APSIM such as phenology and soil moisture, can serve as engineered features in machine learning frameworks, reducing prediction errors in wheat (Feng et al., 2020) and maize (Shahhosseini et al., 2021). Similar integrations in soybean (Corrales et al., 2022) and maize (Zhang et al., 2021), improved the prediction performance by combining environmental data with crop growth model outputs into linear regression models. These integrative models tend to be more transparent, since the process-based component ties predictions to biophysical crop responses, and the data-driven component can quantify feature importance. A second route builds neural experiments that approximate the entire CERES or APSIM parameter surface. Some field-focused studies (McCormick et al., 2021; Xiao et al., 2022; Droutsas et al., 2022; Cunha et al., 2023) reinforced that coupling data-driven and process-based techniques provides more interpretable agronomic adjustments under climate adaptation scenarios. Likewise, Li et al. (2023); Gallear (2023) and Chang et al. (2023) report that machine learning emulators of crop models enabled faster simulations and more efficient scenario analyses. These tools facilitate real-time exploration of “what-if” management decisions and provide interpretable outputs. Overall, these studies highlight that integrating knowledge from the process-based models domain with the flexibility of machine learning, results in more accurate and data-efficient models that are also transparent and actionable advancing decision making for breeders, agronomists, and farmers.

This paper presents a novel data-driven crop model for biomass sorghum growth simulation. The model integrates a descriptive sorghum growth framework—tracking phenotypic responses to genotype, environment, and management (G×E×M) interactions—with data-driven calibration of genotypic parameters from experimental data. Unlike conventional process-based models that treat genotypes as fixed inputs, our approach explicitly disentangles G×E×M effects on phenotypes during the sorghum growth stage by parameterizing genetic properties for each genotype. This methodology streamlines the calibration of complex coefficients inherent in process-based models and reduces reliance on uncertain parameters derived from field experiments, which are often confounded by G×E×M interactions. Additionally, our modular framework adapts to data availability, eliminating the need for predetermined datasets or assumptions about missing information. This adaptability stands in contrast to traditional models, which require extensive data imputation prior to implementation. To the best of our knowledge, this paper presents the first attempt to merge a crop model and a data-driven model to address biomass sorghum yield prediction.

## 2 Materials and methods

In this section, we first present the input data used in this study, then demonstrate the sorghum growth model used in the data-driven crop model approach, followed by the training approach.

### 2.1 Sorghum data

Sorghum phenotypic data were collected from field trials conducted in 2021 and 2022 at the Iowa State University Agricultural Engineering and Agronomy farm, in Boone, IA. The experiments were conducted using a randomized complete blocks design with two replications, with a planting rate of 12 pl/*m*^2^ and 70 cm inter-row spacing. The trials evaluated the Photoperiod Sensitive Panel, which includes 270 photoperiod sensitive (PS) sorghum genotypes (Yu et al., 2016). PS sorghum requires a daylength shorter than 12 hours and 20 minutes for flowering (Rooney and Aydin, 1999), and is primarily cultivated for biomass production. Its extended vegetative stage in temperate and subtropical climates results in higher total dry biomass of leaves and stems compared to other sorghum types during later growth phases (Rooney and Aydin, 1999; Hao et al., 2014). The dataset includes phenotypic records for 11 time points during the growing season (22–145 days after planting), along with genotype and management data. Although the exact sampling dates differed by one or two days, the scheduled measurement points were 22, 36, 43, 50, 57, 64, 71, 78, 85, 110, and 145 days after planting. Phenotypic measurements included dry biomass weights of stems and leaf blades. Each fraction comprises the total biomass of the main culm and, where present, the tillers. Dry biomass was recorded after drying the samples at 60 °C until constant weight. Additionally, management records were collected, containing information on planting date, harvest date, and stand count (plant population density).

### 2.2 Weather data

To comprehensively account for the effect of weather on sorghum growth, weather data was retrieved from the Iowa Environmental Mesonet Herzmann and Wolt (2020), which includes an automated weather station at the farm where the experiments were performed. The variables obtained were air temperature, relative humidity, solar radiation, precipitation, wind speed, evapotranspiration, soil temperature (at 4, 12, 24, 50 inch depth), and soil volumetric water content (at 12, 24, 50 inch depth).

### 2.3 Sorghum growth model

**Figure 1:**
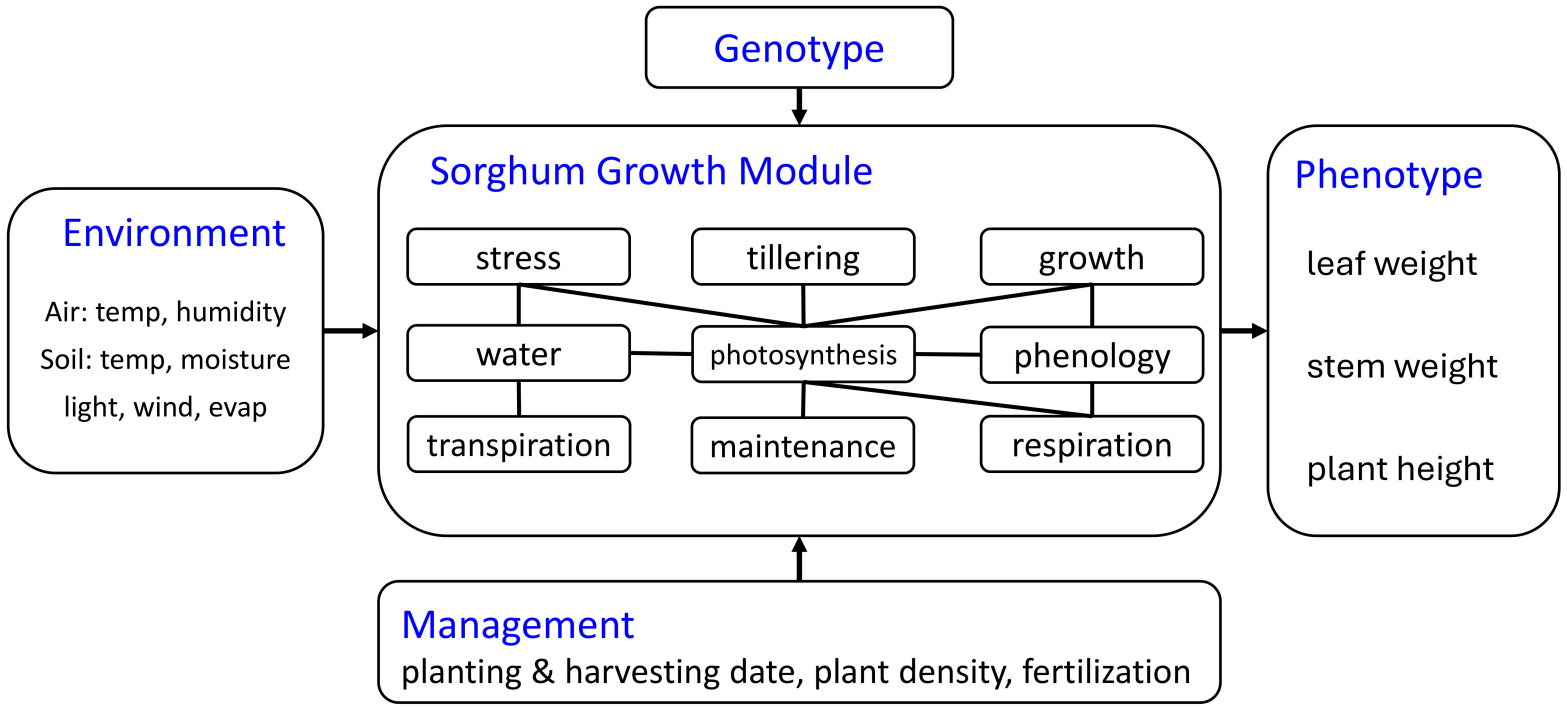
Architecture of the proposed sorghum growth process simulation model.

Our sorghum growth model is designed based on the available data previously described. It has a customized module structure adapted to the modeling granularity provided by the available data. Although the crop model includes a grain component, PS sorghum does not produce grain in temperate environments. In this paper, we focus on tracking the phenotype during the sorghum growth process, with particular emphasis on total biomass weight, which is determined by the dry weight of leaves and stems. Based on these considerations, we constructed a sorghum growth model using the module structure shown below (**Figure 1**). More detailed definitions and equations are illustrated in **Supplementary Presentation 1**.

- Stress: heat and cold stresses based on air temperature and root temperature are considered. Air stress can influence leaf, stem, and root stress only affects the root.
- Tillering: tillers have their own leaf, stem, grain, and root systems.
- Growth: the model updates leaf weight, root weight, root length, stem weight, stem height, grain weight, after considering maintenance and growth respiration. Root length and stem height do not decrease due to irreversible cell expansion and lignification. Under carbon deficit (maintenance *>* growth), biomass is remobilized from existing pools (weight decline) while maintaining structural dimensions, reducing tissue density. When growth biomass is replenished, the model first restores tissue density before allocating to new growth.
- Water: water can be stored and transported in the xylem of the main crop and tiller. Plant water uptake is influenced by root system efficiency and xylem transport capacity, while stand count affects water availability through competition among neighboring plants. Soil water volume is treated as an external input, independent of plant activity since it is considered an input data.
- Photosynthesis: the daily biomass accumulation is determined by light, water, leaf, and phloem capacity constraints, radiation interception is modeled as a function of stand count.
- Phenology: there are four transition points for the sorghum growth in our model, 1) planting, 2) vegetative stage, 3) bloom and grain filling, and 4) harvest. The phenology module does not include stage 3 (bloom and grain filling) for PS sorghum.
- Transpiration: air temperature, humidity, evaporation, and wind can affect the transpiration.
- Maintenance: the amount of photosynthate consumption for maintenance and senescence for leaf, root, stem, and grain, determined by organ weight and stress.
- Respiration: the respiration consumes the photosynthate and provides energy for plant growth activity.

Our data-driven crop model approach can separate input data, output data, genotype-specific properties, intermediate variables, and output variables. Other crop models like APSIM and DSSAT use parameters that are jointly determined by genotype and environment interactions. Instead of using growing degree days (GDD) as the threshold for growing stage transitions and biomass partition ratio, we define a growing degree unit (GDU) in eq. (*S*2) which is similar to GDD but is determined by hourly temperature. The GDU has more capability to capture weather fluctuations on an hourly temporal scale instead of being potentially misleading like average scale data. We also define a growing phenology unit (GPU) in eq (*S*3) and (*S*4) which is calculated by normalized temperature and normalized solar radiation to determine the growth stage. The data-driven crop approach calibrates the parameters using data rather than using predetermined coefficients. This difference provides advantages including compatibility with state-of-the-art data-driven calibration algorithms and adaptability with breeding algorithms.

### 2.4 Training approach

To demonstrate the effectiveness of the data-driven crop approach, we applied the sorghum growth model to the dataset described previously. Computational experiments were conducted using Python on the High Performance Computing Center at Oklahoma State University with dual Intel “Skylake” 6130 CPUs 192 2.1GHz and 96 GB RAM. The data-driven training method is illustrated below.

The missing data in the weather dataset were imputed using the k-nearest neighbors (kNN) method (Fu et al., 2019; Hamzah et al., 2021), which is widely used to handle missing values in crop-yield prediction studies. Genotypic parameters are seeded with values taken from APSIM 7.10 (Holzworth et al., 2014) together with expert-derived bounds, providing a biologically plausible starting point that speeds convergence. Following the workflow described in Section 2.4 (**Algorithm 1**), we trained the model on one experimental year and validated it on the other. The calibration of genotypic parameters g∗ uses the relative root mean square error (RRMSE) as the performance metric, which scales the classic RMSE by the mean observed value, making it easier to compare across traits and years. The heuristic algorithm applies an iterative search: 1) randomly select n parameters from the N-dimensional vector g∗, assigning higher sampling probability to parameters with greater local sensitivity, 2) for each selected parameter, propose two new values: one incremented and one decremented by the current step size, 3) evaluate current RRMSE for each proposed g∗ vector, 4) update the optimal g∗ if a proposal yields better RRMSE, otherwise retain the current solution and proceed to the next iteration. The search terminates when the time limit is reached or when the RRMSE falls below a predefined tolerance. The function GetRRMSE calculates the RRMSE of dry biomass from the given training set. The RRMSE can be calculated as:


$$RRMSE=\frac{\sqrt{\frac{1}{n}\sum_{i=1}^{n}{\left(x_{i,t}-{\hat{x}}_{i,t}\right)}^{2}}}{\frac{1}{n}\sum_{i=1}^{n}x_{i,t}}$$

where,

-
n, sample size number.
-
$x_{i,t}$, the observed total dry biomass weight of leaves and stems in sample i on day t.
-
${\hat{x}}_{i,t}$ the predicted total dry biomass weight of leaves and stems in sample i on day t.

**Algorithm 1:**
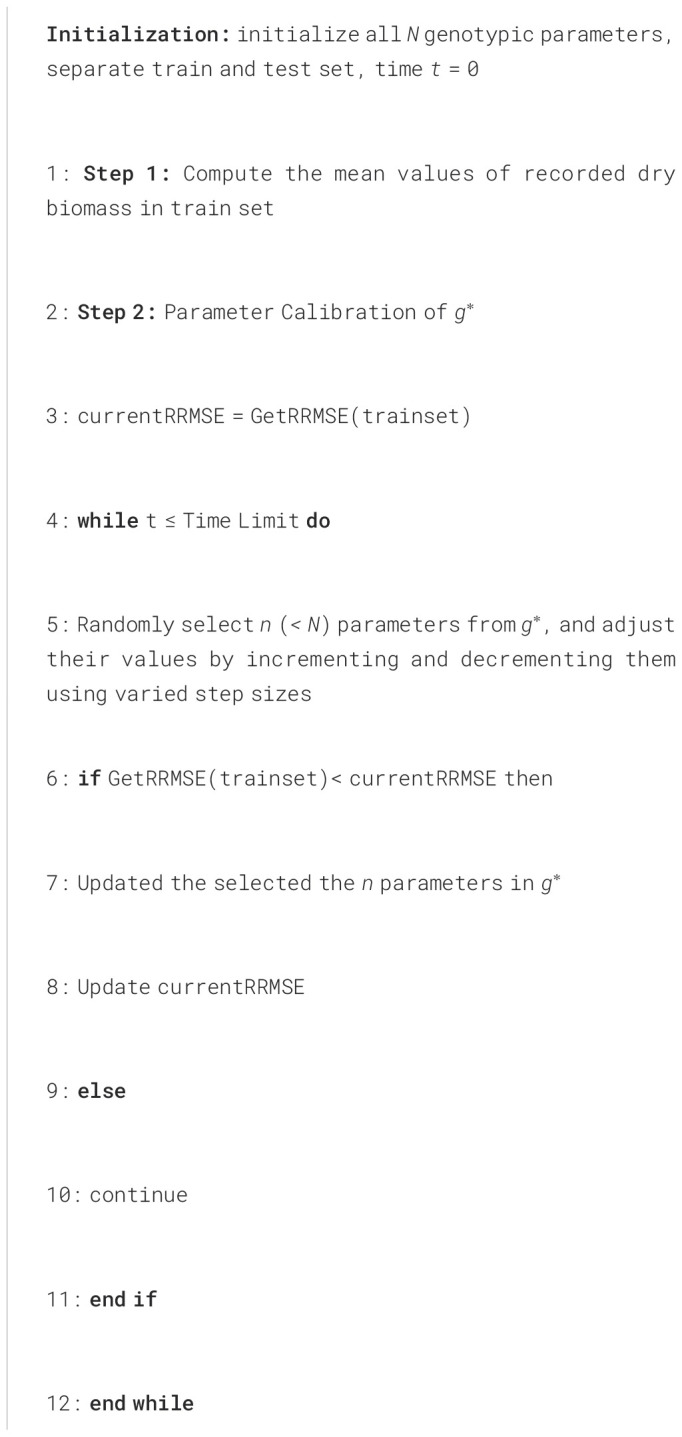
Heuristic Algorithm for Tuning Genotypic Parameters.

## 3 Results

In this section, we demonstrate the training and test strategies and results of our data-driven crop model, followed by additional noteworthy findings.

### 3.1 Phenotypic data

**Figure 2:**
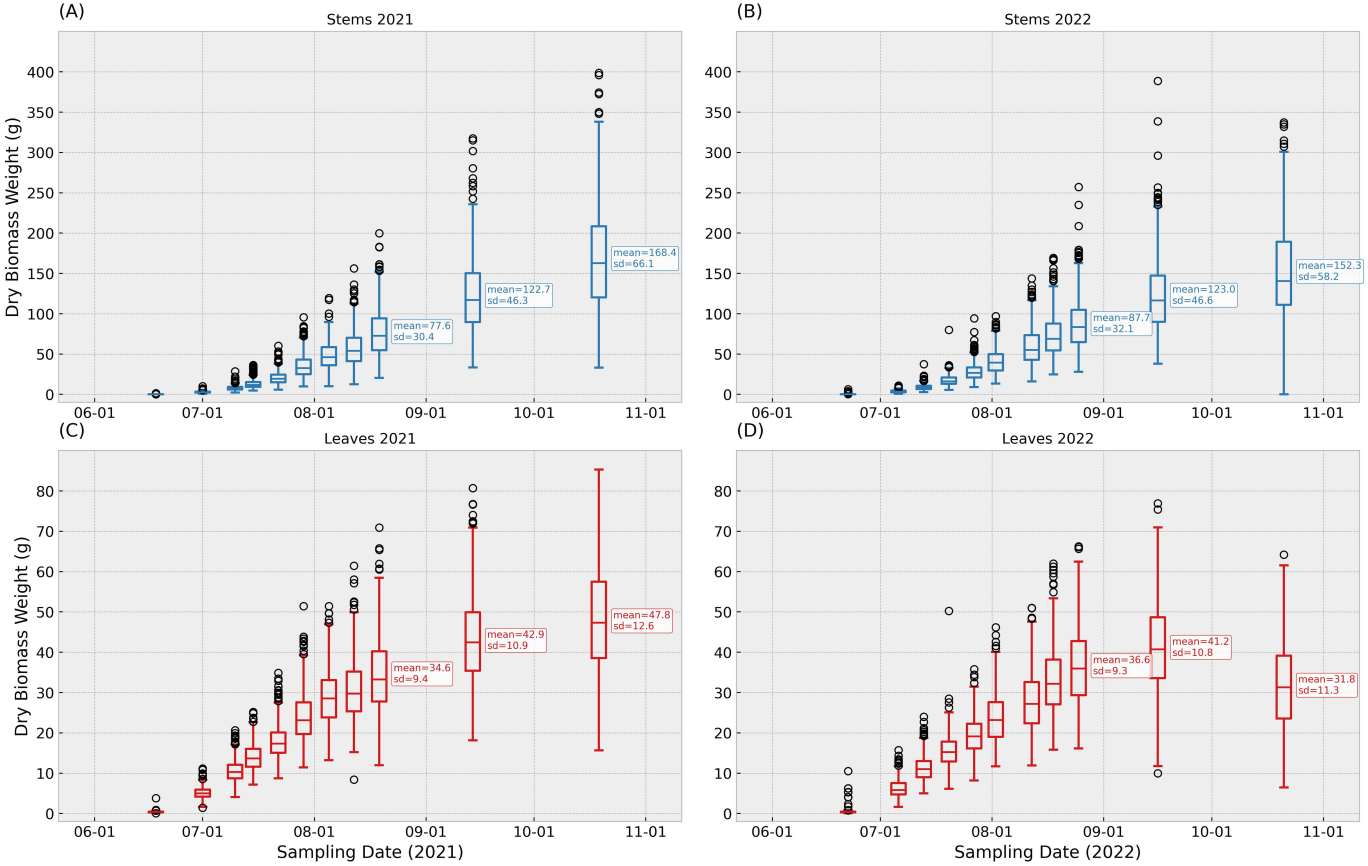
Distribution of dry biomass for stems (blue) and leaves (red) from field trials in Boone, IA, in 2021 and 2022. Panels correspond to 11 scheduled sampling points at 22, 36, 43, 50, 57, 64, 71, 78, 85, 110, and 145 days after planting; calendar dates occasionally differed by 1–2 days. Boxes show the interquartile range (IQR) with the median line; whiskers extend to 1.5×IQR; circles denote observations outside the whiskers. Each observation represents one plant per organ at that date. To aid interpretation, the last three sampling points in each panel are annotated with the sample mean and standard deviation. **Figure 2****(A)** contains stems records in 2021, **(B)** contains stems records in 2022, **(C)** contains leaves records in 2021, **(D)** contains leaves records in 2022.

**Figure 2** displays box plots of leaves and stems dry weights for the two field trials carried out in Boone, IA. Each pair of leaves and stems dry biomass weights box-plots represents the corresponding phenotypic data characterized the same date. By the end of the growing season, stems biomass showed larger values and variability compared to the leaves biomass. Both experiments displayed a consistent increase in biomass accumulation, with differences between years. Notoriously, the leaves fraction showed a lower biomass at the end of the season in 2022, compared to the same sampling point in 2021.

### 3.2 Environmental characterization

**Figure 3:**
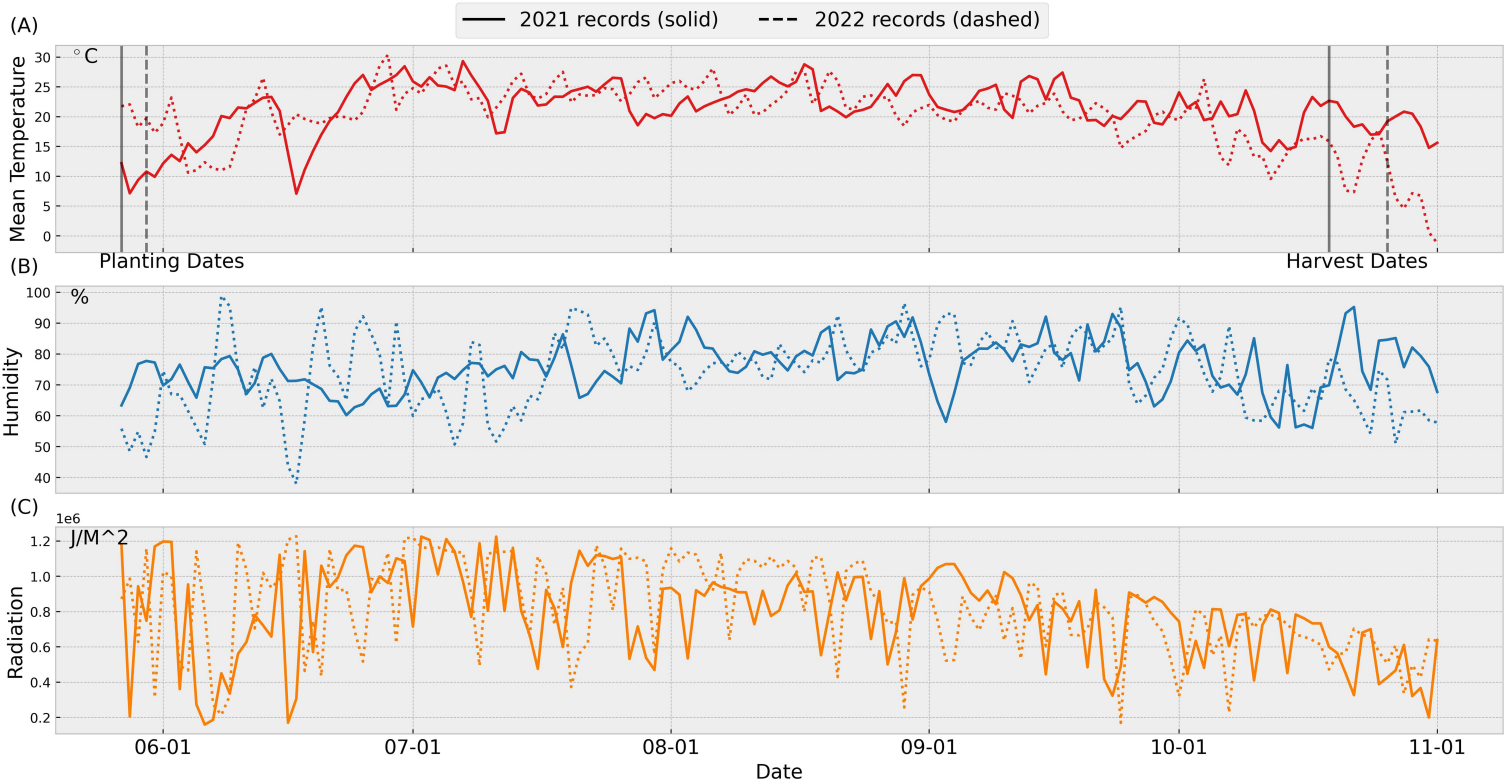
Weather data for 2021 and 2022: The weather data are retrieved from the Iowa Environmental Mesonet Herzmann and Wolt (2020). The data was collected from the automated weather station nearby the trial field location at the Iowa State University Agricultural Engineering and Agronomy farm, in Boone, IA. The planting and harvest dates in 2021 (2022) are 5/27 (5/30) and 10/17 (10/26), respectively. **Figure 3****(A)** temperature, **(B)** humidity, **(C)** radiation.

In 2021, conditions for planting and crop establishment were excellent. In 2022, although temperatures were initially higher than in 2021, there was a very heavy rain after planting, which had a negative impact on seed germination. **Figure 3** summarizes weather conditions during the 2021 and 2022 growing seasons. Notably, mean temperatures in October 2022 were significantly lower than those during the same period in 2021. This temperature anomaly aligns with observed reductions in leaves dry biomass weights near mid-October in 2022.

### 3.3 Training and test results

**Figure 4:**
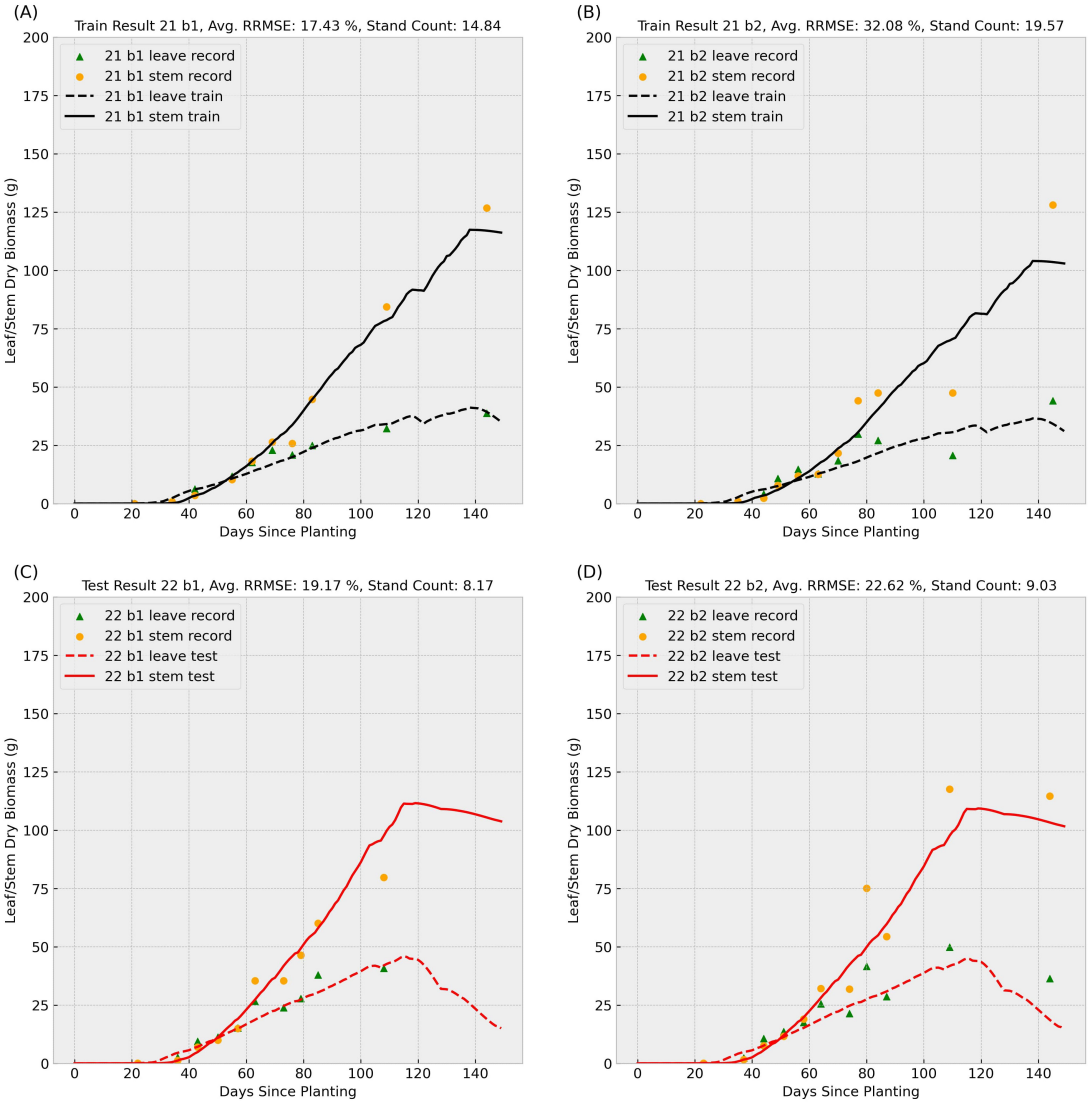
Sample result 1 (Training with 2021 Data): This figure shows the model’s performance when trained on 2021 records for genotype ID 156510 and tested on the same genotype’s 2022 data. Scatter points represent observed leaf and stem dry biomass weights across the growing season, while solid and dashed lines indicate the predicted stem and leaf biomass weights, respectively. Labels “b1” and “b2” denote biological replicate numbers in the field trial. The stand counts reflected in the four observed data series are also included in each small title. The upper **(A, B)** subplots summarize training results for block 1 and 2 in 2021, and the lower **(C, D)** subplots illustrate test performance for block 1 and 2 in 2022.

**Figure 5:**
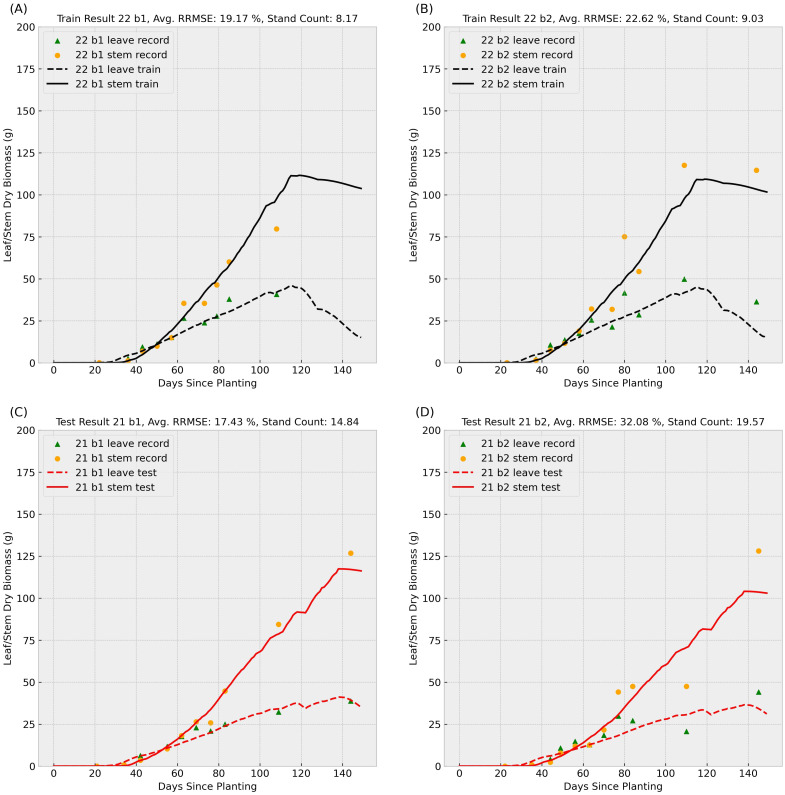
Sample result 2 (Training with 2022 Data): This figure shows the model’s performance when trained on 2022 records for genotype ID 156510 and tested on the same genotype’s 2021 data. Scatter points represent observed leaf and stem dry biomass weights across the growing season, while solid and dashed lines indicate the predicted stem and leaf biomass weights, respectively. Labels “b1” and “b2” denote biological replicate numbers in the field trial. The stand counts reflected in the four observed data series are also included in each small title. The upper **(A, B)** subplots summarize training results for block 1 and 2 in 2022, and the lower **(C, D)** subplots illustrate test performance for block 1 and 2 in 2021.

After masking missing values, the refined dataset consisted of 265 genotypes, with each genotype having two replicates per year at varying stand counts (plants per square meter). This resulted in 530 series of leaves and stems dry biomass weight measurements annually. We conducted two train-test experiments: (1) training on 2021 data and testing on 2022 data, and (2) training on 2022 data and testing on 2021 data. **Figures 4**, **5** summarize two sample results for the same genotype after applying the data-driven crop model to the training and test datasets.

In the **Figures 4**, **5**, scatter points represent observed leaves and stems dry biomass weights across the growing season, while solid and dashed lines denote predicted stems and leaves dry biomass, respectively. Labels “b1” and “b2” indicate replication number in the randomized complete block design used in the field trial, following with varying stand counts across the four observed data series. Training results (upper subplots) generally exhibit lower Relative Root Mean Square Errors (RRMSEs) compared to test results (lower subplots), a common outcome as models are optimized for training data. We can also observe that the data-driven crop model can provide an accurate prediction of sorghum dry biomass production with unseen weather data.

Training RRMSEs were similar across experiments (approximately 20%), whereas test RRMSEs were significantly higher (**Table 1**), suggesting potential overfitting. We also conducted an additional experiment training the model on combined 2021 and 2022 data; results are presented in the final row of **Table 1**. To further analyze parameter behavior, **Supplementary Figure S1** illustrates distinct probability density curves for 56 parameters under three training scenarios: (1) 2021 dataset, (2) 2022 dataset, and (3) combined dataset.

**Table 1:** Training and test performance for different training sets.

Train on 2021 and test on 2022	Train avg. RRMSE	Test avg. RRMSE
Mean	19.93%	40.45%
Standard Deviation	4.62%	16.93%
Train on 2022 and test on 2021	Train avg. RRMSE	Test avg. RRMSE
Mean	20.39%	51.07%
Standard Deviation	6.73%	26.26%
Train on both 2021 and 2022	Train avg. RRMSE	
Mean	23.19%	
Standard Deviation	5.43%	

### 3.4 Changing stand counts

**Figure 6:**
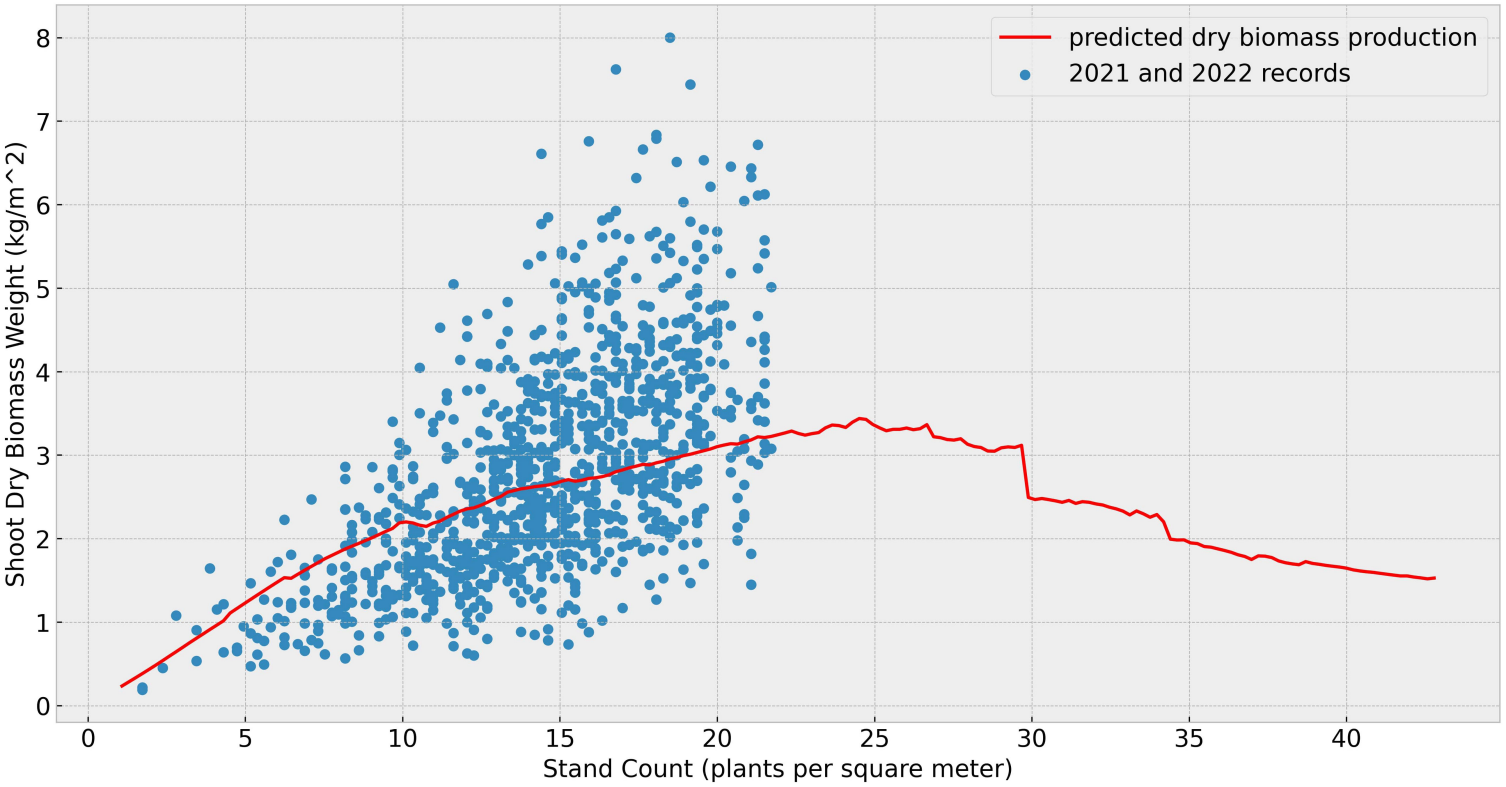
Dry biomass under different stand counts: The blue dots represent the total shoot dry biomass (in kilograms per square meter), calculated from the observed final shoot dry biomass per plant and the stand counts. The red line indicates the simulated shoot dry biomass under varying densities but the same growing environment in 2021.

In this subsection, we conducted a series of simulations to identify the optimal stand count for maximizing biomass production. The stand counts in the training data have a mean value of 14.56 pl/ $m^{2}$ with the standard deviation of 3.83. The simulations were conducted with genotypic parameters calibrated using data from both 2021 and 2022, and assumptions of same weather conditions in 2021 with same soil moisture levels. **Figure 6** compares simulated biomass yields (red line) against observed 2021 and 2022 field data (blue dots). The highest biomass yield based on the simulation was observed at approximately 25 pl/ $m^{2}$, with dry biomass production reaching 3.2 kg/ $m^{2}$. The sudden drop in shoot biomass around 30 pl/ $m^{2}$ is likely due to environmental conditions not represented in the training data. While a comprehensive optimal density analysis will require further field validation environment to confirm these outputs, the present density tests still yield valuable insights and underscore the model’s potential for prescriptive analysis despite limited training data.

### 3.5 Changing planting and harvest dates

**Figure 7:**
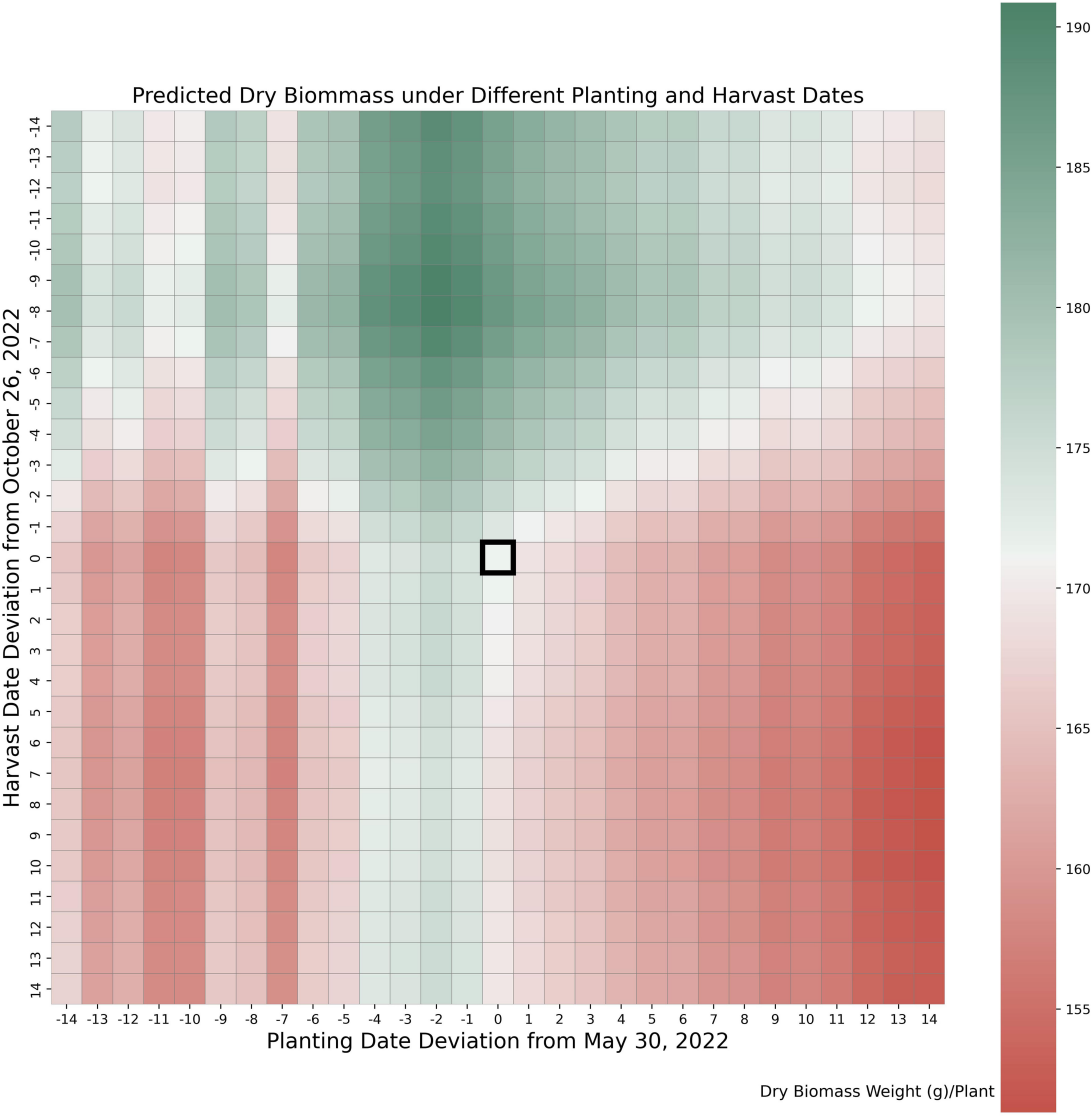
Yield under varying planting and harvesting dates. Values in horizontal and vertical axes indicate numbers of days deviation from actual planting date (May 30) and harvesting date (November 26) in 2022.

We conducted a series of tests to evaluate whether the original planting and harvest dates were optimal under 2022 weather conditions, using parameters calibrated with data from both years. The original planting and harvest dates for the 2022 trial were May 30th and October 26th, respectively. As shown in **Figure 7**, these dates were suboptimal. Shifting the planting date 1–2 days earlier and the harvest date 8–9 days earlier would maximize yield. The simulated peak shootdry biomass is about 9% higher than the original value, with most of the increase contributed by the leaves. This adjustment aligns with the weather patterns illustrated in **Figure 3**, where early harvesting helped avoid severe cold stress observed in late October. Cold stress during this period can accelerate leaf senescence, leading to significant dry biomass loss.

## 4 Discussion

Our data-driven crop model for biomass sorghum demonstrated robust predictive performance, achieving an average Relative Root Mean Square Error (RRMSE) of approximately 20% across experiments trained on the 2021 and 2022 datasets. The model’s training performance is comparable to contemporary crop biomass prediction frameworks (Roy Choudhury et al., 2021; Servia et al., 2022), but underperforms relative to yield prediction models in agricultural applications (Jégo et al., 2012; Xu et al., 2020; Roy Choudhury et al., 2021; Khaki et al., 2021; Dhillon et al., 2023; Chang et al., 2023). However, elevated RRMSE values in test results suggest potential overfitting, likely attributed to limited data availability for each genotype. Furthermore, the model’s accurate prediction of post-120-day leaves dry biomass trends in both years demonstrates its capacity to distinguish genotypic and environmental influences. By isolating the impacts of genotype, environment, and management, the model offers actionable insights for both descriptive analysis and prescriptive agricultural optimization.

Yields at various stand counts can provide critical insights for farmers seeking to maximize profits. Our results indicate that higher stand counts does not ensure increased biomass production, a finding consistent with prior studies (Turgut et al., 2005; Snider et al., 2012; Adams et al., 2015; May et al., 2015; Mahmood et al., 2015; Xuan et al., 2015; Tang et al., 2018). While the literature suggests that the optimal biomass production for sorghum typically occurs at 10–20 *pl/m*^2^, our simulation results exceed this range (Snider et al., 2012; Adams et al., 2015; May et al., 2015; Xuan et al., 2015; Tang et al., 2018). This discrepancy may be attributed to idealized assumptions in our model, such as soil moisture and nutrient availability, which could elevate the optimal stand count. Due to current data limitations, our model does not incorporate seed or labor costs during the sorghum growing process. However, we emphasize that the model’s flexible framework allows for seamless integration of these variables once additional data become available, enabling future analyses with alternative objective functions (e.g., cost-benefit optimization).

The results from planting and harvest dates adjustments test suggests yield improvements harvesting 8 days earlier. The results from the different planting and harvest dates tested indicate that the potential value of data-driven crop models for prescriptive analysis would not have been possible without their ability to separate the genotypic and environmental effects of crop yield. Separating these influences is a crucial feature that enables the data-driven crop model to provide useful recommendations and insights for optimizing crop planting practices. Note that the current model has limited capability to capture certain real-world risks associated with earlier or later planting and harvesting. These include poor emergence due to cold soil temperatures, insect damage linked to delayed planting, or frost risks resulting from late harvesting.

By parameterizing the genotypic properties, our model circumvents calibration challenges inherent to conventional process-based approaches. The proposed data-driven crop model has the ability to fundamentally distinguish genotypic and environmental effects on crop yield, which can unlock valuable prescriptive potential. After obtaining a set of explainable and insightful results, the parameters from our model are transferable to other environments, whereas the genotype parameters for other process-based crop models may need to be recalibrated when the same varieties are grown in different environments (Adnan et al., 2019; Chang et al., 2023; Shawon et al., 2024; Wallach et al., 2025). Such advantages could empower farmers to optimize planting schedules using weather forecasts, reducing reliance on costly field trials for parameter recalibration. Simulations that combine weather forecasts with our model could help farmers choosing sowing dates that favor seed germination, promoting even crop emergence and biomass accumulation and allowing the crop to take advantage of a favorable growing season. If conditions appear unfavorable, the model can recommend delaying planting or scheduling a second sowing to maximize yield. Likewise, toward the end of the growing period, integrating our model with real-time forecasts could alert farmers to the risk of a killing frost, enabling timely harvest and preventing the potential biomass and sugar losses that can follow a sudden cold snap. Our data-driven model has modular flexibility, allowing adaptation to data availability without requiring imputation or assumptions for missing inputs. This adaptability streamlines model development for diverse datasets.

The proposed data-driven model has limitations. The modular structure for one crop species is not easily transferable to another, as each crop has unique physiological properties that need a carefully re-designed framework suitable to that specific species’ biology and growth processes. In addition, the performance is heavily dependent on the quality and quantity of input data. Furthermore, some key practices of management like irrigation, fertilization, and tilling methods are absent in the current version of data-driven crop model.

The results of applying this data-driven model in biomass sorghum could lead to additional data-integration strategies. First, results from our model may provide insightful information that can be readily adapted to other sorghum types as well. Second, UAV and remote sensing data could be incorporated into the model to provide a more comprehensive framework for crop growth. Third, other phenotypic data such as leaf temperature and root depth can be integrated within the data-driven crop model to achieve more reliable simulation and yield prediction results. Furthermore, the data-driven modeling framework could be applied to more crop species and even more complex systems.

## Data Availability

The raw data supporting the conclusions of this article will be made available by the authors, without undue reservation.

## References

[B1] Adams C. B. Erickson J. E. Campbell D. N. Singh M. P. Rebolledo J. P. (2015). Effects of row spacing and population density on yield of sweet sorghum: Applications for harvesting as billets. Agron. J. 107, 1831–1836. doi: 10.2134/agronj14.0295

[B2] Adnan A. A. Diels J. Jibrin J. M. Kamara A. Craufurd P. Shaibu A. . (2019). Options for calibrating ceres-maize genotype specific parameters under data-scarce environments. PLoS One 14, e0200118. doi: 10.1371/journal.pone.0200118, PMID: 30779756 PMC6380597

[B3] Alibabaei K. Gaspar P. D. Lima T. M. Campos R. M. Girão I. Monteiro J. . (2022). A review of the challenges of using deep learning algorithms to support decision-making in agricultural activities. Remote Sens. 14, 638. doi: 10.3390/rs14030638

[B4] Biazin B. Sterk G. Temesgen M. Abdulkedir A. Stroosnijder L. (2012). Rainwater harvesting and management in rainfed agricultural systems in sub-saharan africa–a review. Phys. Chem. Earth Parts A/B/C 47, 139–151. doi: 10.1016/j.pce.2011.08.015

[B5] Boatwright J. L. Brenton Z. W. Boyles R. E. Sapkota S. Myers M. T. Jordan K. E. . (2021). Genetic characterization of a sorghum bicolor multiparent mapping population emphasizing carbon-partitioning dynamics. G3 11, jkab060. doi: 10.1093/g3journal/jkab060, PMID: 33681979 PMC8759819

[B6] Boatwright J. L. Sapkota S. Myers M. Kumar N. Cox A. Jordan K. E. . (2022). Dissecting the genetic architecture of carbon partitioning in sorghum using multiscale phenotypes. Front. Plant Sci. 13, 790005. doi: 10.3389/fpls.2022.790005, PMID: 35665170 PMC9159972

[B7] Breitzman M. W. Bao Y. Tang L. Schnable P. S. Salas-Fernandez M. G. (2019). Linkage disequilibrium mapping of high-throughput image-derived descriptors of plant architecture traits under field conditions. Field Crops Res. 244, 107619. doi: 10.1016/j.fcr.2019.107619

[B8] Brenton Z. W. Cooper E. A. Myers M. T. Boyles R. E. Shakoor N. Zielinski K. J. . (2016). A genomic resource for the development, improvement, and exploitation of sorghum for bioenergy. Genetics 204, 21–33. doi: 10.1534/genetics.115.183947, PMID: 27356613 PMC5012387

[B9] Chang Y. Latham J. Licht M. Wang L. (2023). A data-driven crop model for maize yield prediction. Commun. Biol. 6, 439. doi: 10.1038/s42003-023-04833-y, PMID: 37085696 PMC10121691

[B10] Chen K. O’Leary R. A. Evans F. H. (2019). A simple and parsimonious generalised additive model for predicting wheat yield in a decision support tool. Agric. Syst. 173, 140–150. doi: 10.1016/j.agsy.2019.02.009

[B11] Chimonyo V. Modi A. Mabhaudhi T. (2016). Simulating yield and water use of a sorghum–cowpea intercrop using apsim. Agric. Water Manage. 177, 317–328. doi: 10.1016/j.agwat.2016.08.021

[B12] Conradt T. Gornott C. Wechsung F. (2016). Extending and improving regionalized winter wheat and silage maize yield regression models for Germany: enhancing the predictive skill by panel definition through cluster analysis. Agric. For. Meteorology 216, 68–81. doi: 10.1016/j.agrformet.2015.10.003

[B13] Corrales D. C. Schoving C. Raynal H. Debaeke P. Journet E.-P. Constantin J. (2022). A surrogate model based on feature selection techniques and regression learners to improve soybean yield prediction in southern France. Comput. Electron. Agric. 192, 106578. doi: 10.1016/j.compag.2021.106578

[B14] Cunha R. L. d. F. Silva B. Avegliano P. B. (2023). A comprehensive modeling approach for crop yield forecasts using ai-based methods and crop simulation models. arXiv preprint arXiv:2306.10121. doi: 10.48550/arXiv.2306.10121

[B15] Della Nave F. N. Ojeda J. J. Irisarri J. G. N. Pembleton K. Oyarzabal M. Oesterheld M. (2022). Calibrating apsim for forage sorghum using remote sensing and field data under sub-optimal growth conditions. Agric. Syst. 201, 103459. doi: 10.1016/j.agsy.2022.103459

[B16] Dhillon M. S. Dahms T. Kuebert-Flock C. Rummler T. Arnault J. Steffan-Dewenter I. . (2023). Integrating random forest and crop modeling improves the crop yield prediction of winter wheat and oil seed rape. Front. Remote Sens. 3, 1010978. doi: 10.3389/frsen.2022.1010978

[B17] Drees L. Demie D. T. Paul M. R. Leonhardt J. Seidel S. J. Döring T. F. . (2024). Data-driven crop growth simulation on time-varying generated images using multi-conditional generative adversarial networks. Plant Methods 20, 93. doi: 10.1186/s13007-024-01205-3, PMID: 38879522 PMC11179353

[B18] Droutsas I. Challinor A. J. Deva C. R. Wang E. (2022). Integration of machine learning into process-based modelling to improve simulation of complex crop responses. silico Plants 4, diac017. doi: 10.1093/insilicoplants/diac017

[B19] Duan T. Chapman S. Guo Y. Zheng B. (2017). Dynamic monitoring of ndvi in wheat agronomy and breeding trials using an unmanned aerial vehicle. Field Crops Res. 210, 71–80. doi: 10.1016/j.fcr.2017.05.025

[B20] Feng P. Wang B. Li Liu D. Waters C. Xiao D. Shi L. . (2020). Dynamic wheat yield forecasts are improved by a hybrid approach using a biophysical model and machine learning technique. Agric. For. Meteorology 285, 107922. doi: 10.1016/j.agrformet.2020.107922

[B21] Fu Y. He H. S. Hawbaker T. J. Henne P. D. Zhu Z. Larsen D. R. (2019). Evaluating k-nearest neighbor (k nn) imputation models for species-level aboveground forest biomass mapping in northeast China. Remote Sens. 11, 2005. doi: 10.3390/rs11172005

[B22] Gallear J. W. (2023). Using machine learning and process-based crop modelling for regional scale prediction. University of Leeds Woodhouse Lane Leeds LS2 9JT: University of Leeds.

[B23] Habyarimana E. De Franceschi P. Ercisli S. Baloch F. S. Dall’Agata M. (2020). Genome-wide association study for biomass related traits in a panel of sorghum bicolor and s. bicolor× s. halepense populations. Front. Plant Sci. 11, 551305. doi: 10.3389/fpls.2020.551305, PMID: 33281836 PMC7688983

[B24] Hammer G. L. van Oosterom E. McLean G. Chapman S. C. Broad I. Harland P. . (2010). Adapting apsim to model the physiology and genetics of complex adaptive traits in field crops. J. Exp. Bot. 61, 2185–2202. doi: 10.1093/jxb/erq095, PMID: 20400531

[B25] Hamzah F. B. Hamzah F. M. Razali S. M. Samad H. (2021). A comparison of multiple imputation methods for recovering missing data in hydrological studies. Civil Eng. J. 7, 1608–1619. doi: 10.28991/cej-2021-03091747

[B26] Hao B. Xue Q. Bean B. W. Rooney W. L. Becker J. D. (2014). Biomass production, water and nitrogen use efficiency in photoperiod-sensitive sorghum in the texas high plains. Biomass Bioenergy 62, 108–116. doi: 10.1016/j.biombioe.2014.01.008

[B27] He D. Wang E. Wang J. Robertson M. J. (2017). Data requirement for effective calibration of process-based crop models. Agric. For. meteorology 234, 136–148. doi: 10.1016/j.agrformet.2016.12.015

[B28] Herzmann D. Wolt J. (2020). Iowa state university iowa environmental mesonet. Available online at: https://mesonet.agron.iastate.edu/ASOS/ (Accessed April 15, 2024).

[B29] Holzworth D. P. Huth N. I. deVoil P. G. Zurcher E. J. Herrmann N. I. McLean G. . (2014). Apsim–evolution towards a new generation of agricultural systems simulation. Environ. Model. Software 62, 327–350. doi: 10.1016/j.envsoft.2014.07.009

[B30] Jabed M. A. Murad M. A. A. (2024). Crop yield prediction in agriculture: A comprehensive review of machine learning and deep learning approaches, with insights for future research and sustainability. Heliyon 10(24). doi: 10.1016/j.heliyon.2024.e40836, PMID: 39720079 PMC11667600

[B31] Jégo G. Pattey E. Liu J. (2012). Using leaf area index, retrieved from optical imagery, in the stics crop model for predicting yield and biomass of field crops. Field Crops Res. 131, 63–74. doi: 10.1016/j.fcr.2012.02.012

[B32] Jiang D. Yang X. Clinton N. Wang N. (2004). An artificial neural network model for estimating crop yields using remotely sensed information. Int. J. Remote Sens. 25, 1723–1732. doi: 10.1080/0143116031000150068

[B33] Jones J. Hoogenboom G. Porter C. Boote K. Batchelor W. Hunt L. . (2003). The dssat cropping system model. Eur. J. Agron. 18(3-4), 235–265. doi: 10.1016/S1161-0301(02)00107-7

[B34] Khaki S. Pham H. Wang L. (2021). Simultaneous corn and soybean yield prediction from remote sensing data using deep transfer learning. Sci. Rep. 11, 11132. doi: 10.1038/s41598-021-89779-z, PMID: 34045493 PMC8159996

[B35] Khaki S. Wang L. (2019). Crop yield prediction using deep neural networks. Front. Plant Sci. 10, 452963. doi: 10.3389/fpls.2019.00621, PMID: 31191564 PMC6540942

[B36] Khaki S. Wang L. Archontoulis S. V. (2020). A cnn-rnn framework for crop yield prediction. Front. Plant Sci. 10, 492736. doi: 10.3389/fpls.2019.01750, PMID: 32038699 PMC6993602

[B37] Kivi M. Vergopolan N. Dokoohaki H. (2023). A comprehensive assessment of in *situ* and remote sensing soil moisture data assimilation in the apsim model for improving agricultural forecasting across the us midwest. Hydrology Earth System Sci. 27, 1173–1199. doi: 10.5194/hess-27-1173-2023

[B38] Kugedera A. Nyamadzawo G. Mandumbu R. Nyamangara J. (2022). Potential of field edge rainwater harvesting, biomass transfer and integrated nutrient management in improving sorghum productivity in semi-arid regions: a review. Agroforestry Syst. 96, 909–924. doi: 10.1007/s10457-022-00751-w

[B39] Li L. Zhang Y. Wang B. Feng P. He Q. Shi Y. . (2023). Integrating machine learning and environmental variables to constrain uncertainty in crop yield change projections under climate change. Eur. J. Agron. 149, 126917. doi: 10.1016/j.eja.2023.126917

[B40] Lopez J. R. Erickson J. E. Asseng S. Bobeda E. L. (2017). Modification of the ceres grain sorghum model to simulate optimum sweet sorghum rooting depth for rainfed production on coarse textured soils in a sub-tropical environment. Agric. Water Manage. 181, 47–55. doi: 10.1016/j.agwat.2016.11.023

[B41] MacCarthy D. S. Sommer R. Vlek P. L. (2009). Modeling the impacts of contrasting nutrient and residue management practices on grain yield of sorghum (sorghum bicolor (l.) moench) in a semi-arid region of Ghana using apsim. Field Crops Res. 113, 105–115. doi: 10.1016/j.fcr.2009.04.006

[B42] Mahmood A. Hussain A. Shahzad A. N. Honermeier B. (2015). Biomass and biogas yielding potential of sorghum as affected by planting density, sowing time and cultivar. Pak. J. Bot. 47, 2401–2408.

[B43] Masjedi A. Carpenter N. R. Crawford M. M. Tuinstra M. R. (2019). “ Prediction of sorghum biomass using uav time series data and recurrent neural networks,” in Proceedings of the IEEE/CVF Conference on Computer Vision and Pattern Recognition Workshops.

[B44] Masjedi A. Zhao J. Thompson A. M. Yang K.-W. Flatt J. E. Crawford M. M. . (2018). “ Sorghum biomass prediction using uav-based remote sensing data and crop model simulation,” in IGARSS 2018–2018 IEEE International Geoscience and Remote Sensing Symposium ( IEEE), 7719–7722.

[B45] May A. Souza V. F. d. A. G. Fernandes P. G. (2015). Plant population and row spacing on biomass sorghum yield performance. Ciec. Rural 46, 434–439. doi: 10.1590/0103-8478cr20141133

[B46] McCormick R. F. Truong S. K. Rotundo J. Gaspar A. P. Kyle D. Van Eeuwijk F. . (2021). Intercontinental prediction of soybean phenology via hybrid ensemble of knowledge-based and data driven models. silico Plants 3, diab004. doi: 10.1093/insilicoplants/diab004

[B47] Miftahushudur T. Sahin H. M. Grieve B. Yin H. (2025). A survey of methods for addressing imbalance data problems in agriculture applications. Remote Sens. 17, 454. doi: 10.3390/rs17030454

[B48] Olson S. N. Ritter K. Medley J. Wilson T. Rooney W. L. Mullet J. E. (2013). Energy sorghum hybrids: Functional dynamics of high nitrogen use efficiency. Biomass Bioenergy 56, 307–316. doi: 10.1016/j.biombioe.2013.04.028

[B49] Olson S. N. Ritter K. Rooney W. Kemanian A. McCarl B. A. Zhang Y. . (2012). High biomass yield energy sorghum: developing a genetic model for c4 grass bioenergy crops. Biofuels Bioproducts Biorefining 6, 640–655. doi: 10.1002/bbb.1357

[B50] Panelo J. S. Bao Y. Tang L. Schnable P. S. Salas-Fernandez M. G. (2024). Genetics of canopy architecture dynamics in photoperiod-sensitive and photoperiod-insensitive sorghum. Plant Phenome J. 7, e20092. doi: 10.1002/ppj2.20092

[B51] Panelo J. S. E. M. F. Schnable P. S. Salas-Fernandez M. G. (2025). Crop growth model-enabled genetic mapping of biomass accumulation dynamics in photoperiod sensitive sorghum. Plant Genome. 18.3 (2025): e70111. doi: 10.1002/tpg2.70111, PMID: 40931853 PMC12424023

[B52] Roberts M. J. Braun N. O. Sinclair T. R. Lobell D. B. Schlenker W. (2017). Comparing and combining process-based crop models and statistical models with some implications for climate change. Environ. Res. Lett. 12, 095010. doi: 10.1088/1748-9326/aa7f33

[B53] Rooney W. L. Aydin S. (1999). Genetic control of a photoperiod-sensitive response in sorghum bicolor (l.) moench. Crop Sci. 39, 397–400. doi: 10.2135/cropsci1999.0011183X0039000200016x

[B54] Rosenthal W. Vanderlip R. Jackson B. Arkin G. (1989). Sorkam: A grain sorghum crop growth model. Miscellaneous Publ. (USA), 1669.

[B55] Roy Choudhury M. Das S. Christopher J. Apan A. Chapman S. Menzies N. W. . (2021). Improving biomass and grain yield prediction of wheat genotypes on sodic soil using integrated high-resolution multispectral, hyperspectral, 3d point cloud, and machine learning techniques. Remote Sens. 13, 3482. doi: 10.3390/rs13173482

[B56] Salas Fernandez M. G. Bao Y. Tang L. Schnable P. S. (2017). A high-throughput, field-based phenotyping technology for tall biomass crops. Plant Physiol. 174, 2008–2022. doi: 10.1104/pp.17.00707, PMID: 28620124 PMC5543940

[B57] Salas-Fernandez M. G. Kemp J. (2022). Registration of ia100rps and ia101rps sorghum inbred lines for photoperiod-sensitive biomass hybrids. J. Plant Registrations 16, 465–472. doi: 10.1002/plr2.20199

[B58] Servia H. Pareeth S. Michailovsky C. I. de Fraiture C. Karimi P. (2022). Operational framework to predict field level crop biomass using remote sensing and data driven models. Int. J. Appl. Earth Observation Geoinformation 108, 102725. doi: 10.1016/j.jag.2022.102725

[B59] Shahhosseini M. Hu G. Huber I. Archontoulis S. V. (2021). Coupling machine learning and crop modeling improves crop yield prediction in the us corn belt. Sci. Rep. 11, 1606. doi: 10.1038/s41598-020-80820-1, PMID: 33452349 PMC7810832

[B60] Shawon A. R. Memic E. Kottmann L. Uptmoor R. Hackauf B. Feike T. (2024). Comprehensive evaluation of the dssat-csm-ceres-wheat for simulating winter rye against multi-environment data in Germany. Agron. J. 116, 1844–1868. doi: 10.1002/agj2.21590

[B61] Shook J. Gangopadhyay T. Wu L. Ganapathysubramanian B. Sarkar S. Singh A. K. (2021). Crop yield prediction integrating genotype and weather variables using deep learning. PLoS One 16, e0252402. doi: 10.1371/journal.pone.0252402, PMID: 34138872 PMC8211294

[B62] Silva T. N. Thomas J. B. Dahlberg J. Rhee S. Y. Mortimer J. C. (2022). Progress and challenges in sorghum biotechnology, a multipurpose feedstock for the bioeconomy. J. Exp. Bot. 73, 646–664. doi: 10.1093/jxb/erab450, PMID: 34644381 PMC8793871

[B63] Singh A. Newton L. Schnable J. C. Thompson A. M. (2025). Unveiling shared genetic regulators of plant architectural and biomass yield traits in the sorghum association panel. *Journal of Experimental*. Botany 76, 1625–1643. doi: 10.1093/jxb/eraf012, PMID: 39798149 PMC11981901

[B64] Snider J. L. Raper R. L. Schwab E. B. (2012). The effect of row spacing and seeding rate on biomass production and plant stand characteristics of non-irrigated photoperiod-sensitive sorghum (sorghum bicolor (l.) moench). Ind. Crops Products 37, 527–535. doi: 10.1016/j.indcrop.2011.07.032

[B65] Tang C. Sun C. Du F. Chen F. Ameen A. Fu T. . (2018). Effect of plant density on sweet and biomass sorghum production on semiarid marginal land. Sugar Tech 20, 312–322. doi: 10.1007/s12355-017-0553-3

[B66] Tirfessa A. Getachew F. McLean G. Van Oosterom E. Jordan D. Hammer G. (2023). Modeling adaptation of sorghum in Ethiopia with apsim—opportunities with g× e× m. Agron. Sustain. Dev. 43, 15. doi: 10.1007/s13593-023-00869-w, PMID: 36714044 PMC9873777

[B67] Truong S. K. McCormick R. F. Mullet J. E. (2017). Bioenergy sorghum crop model predicts vpd-limited transpiration traits enhance biomass yield in water-limited environments. Front. Plant Sci. 8, 226145. doi: 10.3389/fpls.2017.00335, PMID: 28377779 PMC5359309

[B68] Turgut I. Bilgili U. Duman A. Acikgoz E. (2005). Production of sweet sorghum (sorghum bicolor l. moench) increases with increased plant densities and nitrogen fertilizer levels. Acta Agriculturae Scandinavica Section B-Soil Plant 55, 236–240. doi: 10.1080/09064710510029051

[B69] Varela S. Pederson T. Bernacchi C. J. Leakey A. D. (2021). Understanding growth dynamics and yield prediction of sorghum using high temporal resolution uav imagery time series and machine learning. Remote Sens. 13, 1763. doi: 10.3390/rs13091763

[B70] Virmani S. Tandon H. L. S. Alagarswamy G. (1989). Modeling the growth and development of sorghum and pearl millet ( International Crops Research Institute for the Semi-Arid Tropics).

[B71] Wallach D. Kim K. S. Hyun S. Buis S. Thorburn P. Mielenz H. . (2025). Evaluating the agmip calibration protocol for crop models; case study and new diagnostic tests. Eur. J. Agron. 168, 127659. doi: 10.1016/j.eja.2025.127659

[B72] Wang D. Bean S. McLaren J. Seib P. Madl R. Tuinstra M. . (2008). Grain sorghum is a viable feedstock for ethanol production. J. Ind. Microbiol. Biotechnol. 35, 313–320. doi: 10.1007/s10295-008-0313-1, PMID: 18214563

[B73] Wang T. Crawford M. M. (2021). “ Multi-year sorghum biomass prediction with uav-based remote sensing data,” in 2021 IEEE International Geoscience and Remote Sensing Symposium IGARSS ( IEEE), 4312–4315.

[B74] Wang T. Crawford M. M. Tuinstra M. R. (2023). A novel transfer learning framework for sorghum biomass prediction using uav-based remote sensing data and genetic markers. Front. Plant Sci. 14, 1138479. doi: 10.3389/fpls.2023.1138479, PMID: 37113602 PMC10126475

[B75] White J. Alagarswamy G. Ottman M. J. Porter C. Singh U. Hoogenboom G. (2015). An overview of ceres–sorghum as implemented in the cropping system model version 4.5. Agron. J. 107, 1987–2002. doi: 10.2134/agronj15.0102

[B76] Xiao L. Wang G. Zhou H. Jin X. Luo Z. (2022). Coupling agricultural system models with machine learning to facilitate regional predictions of management practices and crop production. Environ. Res. Lett. 17, 114027. doi: 10.1088/1748-9326/ac9c71

[B77] Xiong Y. Zhang P. Warner R. D. Fang Z. (2019). Sorghum grain: From genotype, nutrition, and phenolic profile to its health benefits and food applications. Compr. Rev. Food Sci. Food Saf. 18, 2025–2046. doi: 10.1111/1541-4337.12506, PMID: 33336966

[B78] Xu J.-X. Ma J. Tang Y.-N. Wu W.-X. Shao J.-H. Wu W.-B. . (2020). Estimation of sugarcane yield using a machine learning approach based on uav-lidar data. Remote Sens. 12, 2823. doi: 10.3390/rs12172823

[B79] Xuan T. D. Phuong N. T. Khang D. T. Khanh T. D. (2015). Influence of sowing times, densities, and soils to biomass and ethanol yield of sweet sorghum. Sustainability 7, 11657–11678. doi: 10.3390/su70911657

[B80] Yang K.-W. Chapman S. Carpenter N. Hammer G. McLean G. Zheng B. . (2021). Integrating crop growth models with remote sensing for predicting biomass yield of sorghum. silico Plants 3, diab001. doi: 10.1093/insilicoplants/diab001

[B81] Yu X. Li X. Guo T. Zhu C. Wu Y. Mitchell S. E. . (2016). Genomic prediction contributing to a promising global strategy to turbocharge gene banks. Nat. Plants 2, 1–7. doi: 10.1038/nplants.2016.150, PMID: 27694945

[B82] Zha T. Barr A. G. van der Kamp G. Black T. A. McCaughey J. H. Flanagan L. B. (2010). Interannual variation of evapotranspiration from forest and grassland ecosystems in western Canada in relation to drought. Agric. For. Meteorology 150, 1476–1484. doi: 10.1016/j.agrformet.2010.08.003

[B83] Zhang L. Zhang Z. Tao F. Luo Y. Cao J. Li Z. . (2021). Planning maize hybrids adaptation to future climate change by integrating crop modelling with machine learning. Environ. Res. Lett. 16, 124043. doi: 10.1088/1748-9326/ac32fd

