# Supplementary material for: A data-driven crop model for biomass sorghum growth process simulation
Source: Front Plant Sci. 2025 Nov 13;16:1617775. doi: 10.3389/fpls.2025.1617775 (PMC12658319; doi:10.3389/fpls.2025.1617775)
Supplement: Supplementary file 1 [file Presentation1.pdf]

Supplementary Material

1 SORGHUM GROWTH MODEL

Environment data:

- E_d^{AirTemp}: air temperature during day d, in Celsius.
- $E_{d,l}^{\text{SoilTemp}}$: soil temperature at layer l during hour h, in Celsius.
- $E_{d,l}^{\text{SoilMoisture}}$: soil moisture at layer l during day d, in %.
- E_d^{Humidity}: relative humidity during day d, in %.
- E_d^{Light}: solar radiation during hour h, in J/m^2.
- E_d^{Wind}: average wind speed during day d, in km/h.
- E_d^{Evap}: potential evapotranspiration during day d, in mm.

Management data:

- M^{Plant}: planting date
- M^{Harvest}: harvesting date
- M^{Density}: stand count (plants$/m^2$)

Phenotypic data:

- P^{Yield}: total biomass dry weight, in kg/ha
- $P^{\text{LeafWeight}}$: dry leaf weight, in gram
- $P^{\text{StemWeight}}$: dry stem weight, in gram

Genotypic property parameters:

1. $g^{\text{GDURootTemp}}$: weight of average root temperature in the GDU definition, unitless
2. g^{MinTemp}: minimum temperature for GDU and GPU calculation, in Celsius
3. g^{MaxTemp}: maximum temperature for GDU and GPU calculation, in Celsius
4. g^{MaxLight}: maximum radiation for GPU calculation, in J/m^2
5. g^{GPUTemp}: power coefficient for temperature in GPU definition, unitless
6. g^{GPULight}: power coefficient for radiation in GPU definition, unitless
7. g_t^{Stage}: amount of cumulative GDU or GPU to reach stage t, in unitless
8. $g^{\text{AccuAirStressRecovery}}$: accumulated air stress recovery speed, unitless
9. $g^{\text{AccuRootStressRecovery}}$: accumulated root stress recovery speed, unitless
10. $g^{\text{AccuAirStress}}$: coefficient for accumulated air stress calculation, unitless
11. $g^{\text{AccuRootStress}}$: coefficient for accumulated root stress calculation, unitless
12. $g^{\text{WaterPlantDensity}}$: coefficient for water stress affected by stand count, unitless
13. $g^{\text{LightPlantDensity}}$: coefficient for radiation absorption affected by stand count, unitless

14. $g_t^{\text{BestAirTemp}}$: best air temperature for leaf, stem, and grain growth in stage t, in Celsius
15. $g_t^{\text{MaxAirTemp}}$: maximal air temperature for leaf, stem, and grain growth in stage t, in Celsius
16. $g_t^{\text{MinAirTemp}}$: minimal air temperature for leaf, stem, and grain growth in stage t, in Celsius
17. $g_t^{\text{AirHeatPower}}$: power coefficient for air heat stress calculation, unitless
18. $g_t^{\text{AirColdPower}}$: power coefficient for air cold stress calculation, unitless
19. $g_t^{\text{BestRootTemp}}$: best temperature for root growth in stage t, in Celsius
20. $g_t^{\text{MaxRootTemp}}$: maximal temperature for root growth in stage t, in Celsius
21. $g_t^{\text{MinRootTemp}}$: minimal temperature for root growth in stage t, in Celsius
22. $g_t^{\text{RootHeatPower}}$: power coefficient for root heat stress calculation, unitless
23. $g_t^{\text{RootColdPower}}$: power coefficient for root cold stress calculation, unitless

24. $g_t^{\text{Xylem/Leaf}}$: xylem capacity as a percentage of leaf weight in stage t, in %
25. $g_t^{\text{Xylem/Stem}}$: xylem capacity as a percentage of stem weight in stage t, in %
26. $g_t^{\text{RootWaterCap}}$: coefficient for root water uptake capacity with respect to soil moisture in stage t, in gram/%

27. $g_t^{\text{TempTranspiration}}$: coefficient for temperature effect on transpiration in stage t, unitless
28. $g_t^{\text{HumidityTranspiration}}$: coefficient for humidity effect on transpiration in stage t, unitless
29. $g_t^{\text{EvapTranspiration0}}$: coefficient for evaporation effect on transpiration in stage t, unitless
30. $g_t^{\text{EvapTranspiration1}}$: another coefficient for evaporation effect on transpiration in stage t, unitless
31. $g_t^{\text{WindTranspiration0}}$: coefficient for wind effect on transpiration in stage t, unitless
32. $g_t^{\text{WindTranspiration1}}$: another coefficient for wind effect on transpiration in stage t, unitless

33. $g_t^{\text{Photo/Light}}$: photosynthesis production per unit of solar radiation in stage t, in gram/J
34. $g_t^{\text{Photo/Water}}$: photosynthesis production per unit of crop water in stage t, in %
35. $g_t^{\text{PhotoWater/Transpiration}}$: ratio between water used for photosynthesis and water used for transpiration in stage t, in %
36. $g_t^{\text{Photo/Leaf}}$: photosynthesis production per unit of leaf weight in stage t, in %
37. $g_t^{\text{Phloem/Leaf}}$: phloem capacity per unit of leaf weight in stage t, in %
38. $g_t^{\text{Phloem/Stem}}$: phloem capacity per unit of stem weight in stage t, in %
39. $g_t^{\Delta\text{Leaf0}}$: coefficient for maximal amount of leaf growth in stage t, in gram
40. $g_t^{\Delta\text{Leaf1}}$: coefficient for maximal amount of leaf growth in stage t, in %
41. $g_t^{\Delta\text{Root0}}$: coefficient for maximal amount of root growth in stage t, in gram
42. $g_t^{\Delta\text{Root1}}$: coefficient for maximal amount of root growth in stage t, in %
43. $g_t^{\Delta\text{Stem0}}$: coefficient for maximal amount of stem growth in stage t, in gram
44. $g_t^{\Delta\text{Stem1}}$: coefficient for maximal amount of stem growth in stage t, in %
45. $g_t^{\Delta\text{Grain0}}$: coefficient for maximal amount of grain growth in stage t, in gram

46. $g_t^{\triangle \text{Grain1}}$: coefficient for maximal amount of grain growth in stage t, in %

47. g_t^{LeafMS0}: coefficient for leaf maintenance and senescence calculation in stage t, in %
48. g_t^{LeafMS1}: coefficient for leaf maintenance and senescence calculation in stage t, in %
49. g_t^{RootMS0}: coefficient for root maintenance and senescence calculation in stage t, in %
50. g_t^{RootMS1}: coefficient for root maintenance and senescence calculation in stage t, in %
51. g_t^{StemMS0}: coefficient for stem maintenance and senescence calculation in stage t, in %
52. g_t^{StemMS1}: coefficient for stem maintenance and senescence calculation in stage t, in %
53. g_t^{GrainMS0}: coefficient for grain maintenance and senescence calculation in stage t, in %
54. g_t^{GrainMS1}: coefficient for grain maintenance and senescence calculation in stage t, in %

55. $g_t^{\text{RootDensity}}$: root weight density in stage t, in gram/centimeter
56. $g_t^{\text{StemDensity}}$: stem weight density in stage t, in gram/centimeter

Variables:

1. $v_d^{\text{ARootTemp}}$: average root temperature weighted at different layers, in Celsius
2. v_h^{GDUTemp}: conventional GDU for hour h, in Celsius
3. v_h^{GPUTemp}: temperature unit of new GPU for hour h, unitless
4. v_h^{GPULight}: radiation unit of new GPU for hour h, unitless
5. v_d^{GPU}: cumulative GPU by day d, unitless
6. t: the current stage number, unitless
7. $v^{\text{LightDensityIndex}}$: radiation absorption stress index based on stand count, unitless
8. $v^{\text{WaterDensityIndex}}$: water stress index based on stand count, unitless

9. v_d^{AirHeat}: heat stress caused by air temperature on leaf, stem, and grain during day d, in %
10. v_d^{AirCold}: cold stress caused by air temperature on leaf, stem, and grain during day d, in %
11. v_d^{RootHeat}: heat stress caused by root temperature on root during day d, in %
12. v_d^{RootCold}: cold stress caused by root temperature on root during day d, in %
13. $v_d^{\text{AirStress}}$: overall stress on leaf, stem, and grain during day d, in %
14. $v_d^{\text{RootStress}}$: overall stress on root during day d, in %
15. $v_d^{\text{AccuAirStress}}$: accumulated air stress on day d, unitless
16. $v_d^{\text{AccuRootStress}}$: accumulated root stress on day d, unitless
17. $v_d^{\text{AccuAirStressIndex}}$: stress index based on previous not recovered air stress during day d, in %
18. $v_d^{\text{AccuRootStressIndex}}$: stress index based on previous not recovered root stress during day d, in %
19. $v_d^{\text{AccuRootStressIndex}}$: stress index based on previous not recovered root stress during day d, in %

20. $v_d^{\text{MXylemCap}}$: xylem capacity for water storage and transport in main crop during day d, in gram
21. $v_d^{\text{TXylemCap}}$: xylem capacity for water storage and transport in tiller during day d, in gram

22. $v_d^{\text{RootWaterCap}}$: root capacity for water uptake during day d, in gram
23. $v_d^{\text{MWaterUptakeCap}}$: capacity of water uptake by main crop during day d, in gram
24. $v_d^{\text{TWaterUptakeCap}}$: capacity of water uptake by tiller during day d, in gram
25. $v_d^{\text{TotalWaterUptake}}$: total amount of water uptake during day d, in gram
26. $v_d^{\text{MWaterUptake}}$: amount of water uptake by main crop from soil during day d, in gram
27. $v_d^{\text{TWaterUptake}}$: amount of water uptake by tiller crop from soil during day d, in gram
28. v_d^{MWater}: amount of crop water storage in xylem of main crop during day d, in gram
29. v_d^{TWater}: amount of crop water storage in xylem of tiller during day d, in gram

30. $v_d^{\text{TempTranspiration}}$: air temperature effect on transpiration rate during day d, in %
31. $v_d^{\text{HumidityTranspiration}}$: humidity effect on transpiration rate during day d, in %
32. $v_d^{\text{EvapTranspiration}}$: evaporation effect on transpiration rate during day d, in %
33. $v_d^{\text{WindTranspiration}}$: wind effect on transpiration rate during day d, in %
34. $v_d^{\text{MTranspiration}}$: amount of transpiration for main crop during day d, in gram
35. $v_d^{\text{TTranspiration}}$: amount of transpiration for tiller during day d, in gram

36. $v_d^{\text{PhotoLightCap}}$: capacity of photosynthesis constrained by solar radiation during day d, in gram
37. $v_d^{\text{MPhotoWaterCap}}$: crop water constraints for the main crop on photosynthesis production during day d, in gram
38. $v_d^{\text{TPhotoWaterCap}}$: crop water constraints for the tiller on photosynthesis production during day d, in gram
39. $v_d^{\text{MPhotoLeafCap}}$: leaf constraints for the main crop on photosynthesis production during day d, in gram
40. $v_d^{\text{TPhotoLeafCap}}$: leaf constraints for the tiller on photosynthesis production during day d, in gram
41. $v_d^{\text{MPhloemCap}}$: phloem capacity in the main crop for photosynthate storage and transport during day d, in gram
42. $v_d^{\text{TPhloemCap}}$: phloem capacity in the tiller for photosynthate storage and transport during day d, in gram
43. v_d^{MPhoto}: amount of biomass production in the main crop through photosynthesis during day d, in gram
44. v_d^{TPhoto}: amount of biomass production in the tiller through photosynthesis during day d, in gram
45. $v_d^{\text{MLeafGrowthCap}}$: maximal amount of leaf growth for the main crop during day d, in gram
46. $v_d^{\text{MRootGrowthCap}}$: maximal amount of root growth for the main crop during day d, in gram
47. $v_d^{\text{MStemGrowthCap}}$: maximal amount of stem growth for the main crop during day d, in gram
48. $v_d^{\text{MGrainGrowthCap}}$: maximal amount of grain growth for the main crop during day d, in gram
49. $v_d^{\text{MGrowthCap}}$: maximal amount of total growth for the main crop during day d, in gram
50. $v_d^{\text{TLeafGrowthCap}}$: maximal amount of leaf growth for the tiller during day d, in gram
51. $v_d^{\text{TRootGrowthCap}}$: maximal amount of root growth for the tiller during day d, in gram
52. $v_d^{\text{TStemGrowthCap}}$: maximal amount of stem growth for the tiller during day d, in gram
53. $v_d^{\text{TGrainGrowthCap}}$: maximal amount of grain growth for the tiller during day d, in gram

54. $v_d^{\text{TGrowthCap}}$: maximal amount of total growth for the tiller during day d, in gram
55. v_d^{MGrowth}: actual total growth for the main crop during day d, in gram
56. v_d^{TGrowth}: actual total growth for the tiller during day d, in gram
57. v_d^{MBiomass}: amount of main crop biomass reserve in phloem during day d, in gram
58. v_d^{TBiomass}: amount of tiller biomass reserve in phloem during day d, in gram
59. $v_d^{\text{MWaterPhoto}}$: amount of water consumption for photosynthesis in the main crop during day d, in gram
60. $v_d^{\text{TWaterPhoto}}$: amount of water consumption for photosynthesis in the tiller during day d, in gram
61. v_d^{MLeafMS}: leaf maintenance and senescence in the main crop during day d, in gram
62. v_d^{MRootMS}: root maintenance and senescence in the main crop during day d, in gram
63. v_d^{MStemMS}: stem maintenance and senescence in the main crop during day d, in gram
64. v_d^{MGrainMS}: grain maintenance and senescence in the main crop during day d, in gram
65. v_d^{TLeafMS}: leaf maintenance and senescence in the tiller during day d, in gram
66. v_d^{TStemMS}: stem maintenance and senescence in the tiller during day d, in gram
67. v_d^{TGrainMS}: grain maintenance and senescence in the tiller during day d, in gram
68. $v_d^{\text{MLeafWeight}}$: main crop leaf weight during day d, in gram
69. $v_d^{\text{MRootWeight}}$: main crop root weight during day d, in gram
70. $v_d^{\text{MRootLength}}$: main crop total root length during day d, in centimeter
71. $v_{d,l}^{\text{MRootLayerLength}}$: main crop root length at layer l during day d, in centimeter
72. $v_d^{\text{MStemWeight}}$: main crop stem weight during day d, in gram
73. $v_d^{\text{MStemHeight}}$: main crop stem height during day d, in centimeter
74. $v_d^{\text{MGrainWeight}}$: main crop grain weight during day d, in gram
75. $v_d^{\text{MShootWeight}}$: main crop shoot (leaf, stem, and grain) weight during day d, in gram
76. $v_d^{\text{TLeafWeight}}$: tiller leaf weight during day d, in gram
77. $v_d^{\text{TRootWeight}}$: tiller root weight during day d, in gram
78. $v_d^{\text{TRootLength}}$: tiller total root length during day d, in centimeter
79. $v_{d,l}^{\text{TRootLayerLength}}$: tiller root length at layer l during day d, in centimeter
80. $v_d^{\text{TStemWeight}}$: tiller stem weight during day d, in gram
81. $v_d^{\text{TStemHeight}}$: tiller stem height during day d, in centimeter
82. $v_d^{\text{TGrainWeight}}$: tiller grain weight during day d, in gram
83. $v_d^{\text{TShootWeight}}$: tiller shoot (leaf, stem, and grain) weight during day d, in gram
84. v_d^{Yield}: yield, which is main crop and tiller shoot weight on the harvest day, in gram

Phenology Module

$$v_h^{\text{ARootTemp}} = \frac{\sum_l [(v_{d-1,l}^{\text{MRootLayerLength}} + v_{d-1,l}^{\text{TRootLayerLength}}) \cdot E_{h,l}^{\text{SoilTemp}}]}{\sum_l (v_{d-1,l}^{\text{MRootLayerLength}} + v_{d-1,l}^{\text{TRootLayerLength}})} \tag{S1}$$

$$\begin{aligned} v_h^{\text{GDUTemp}} &= \min\{\max\{(1 - g^{\text{GDURootTemp}}) \cdot E_h^{\text{AirTemp}} + \\ &\quad g^{\text{GDURootTemp}} \cdot v_h^{\text{ARootTemp}}, g^{\text{MaxTemp}}\}, g^{\text{MinTemp}}\} \end{aligned} \tag{S2}$$

$$v_h^{\text{GPUTemp}} = v_h^{\text{GDUTemp}} / (g^{\text{MaxTemp}} - g^{\text{MinTemp}}) \tag{S3}$$

$$v_h^{\text{GPULight}} = \min\left\{E_h^{\text{Light}} / g^{\text{MaxLight}}, 1\right\} \tag{S4}$$

$$v_d^{\text{GPU}} = v_{d-1}^{\text{GPU}} + \sum_{h \in H(d)} \left[(v_h^{\text{GPUTemp}})^{g^{\text{GPUTemp}}} \cdot (v_h^{\text{GPULight}})^{g^{\text{GPULight}}} \right] \tag{S5}$$

$$t = \max\{\tau : v_d^{\text{GPU}} \geq g_\tau^{\text{Stage}}\} \tag{S6}$$

Equations (S1) and (S2) calculate the cumulative growing degree units determined by weighted average of air and root temperatures. Equations (S3)-(S5) propose a more general definition of the growing phenology units determined by normalized temperature and normalized solar radiation. Equation (S6) determines the current growth stage, which could be using GDU or GPU.

Stress/Efficiency Module

$$v_d^{\text{AirHeat}} = \min\left\{\frac{\max\{E_d^{\text{AirTemp}} - g_t^{\text{BestAirTemp}}, 0\}}{g_t^{\text{MaxAirGrowthTemp}} - g_t^{\text{BestAirTemp}}}, 1\right\}^{g_t^{\text{AirHeatPower}}} \tag{S7}$$

$$v_d^{\text{AirCold}} = \min\left\{\frac{\min\{E_d^{\text{AirTemp}} - g_t^{\text{BestAirTemp}}, 0\}}{g_t^{\text{MinAirGrowthTemp}} - g_t^{\text{BestAirTemp}}}, 1\right\}^{g_t^{\text{AirColdPower}}} \tag{S8}$$

$$v_d^{\text{RootHeat}} = \min\left\{\frac{\max\{v_d^{\text{ARootTemp}} - g_t^{\text{BestRootTemp}}, 0\}}{g_t^{\text{MaxRootGrowthTemp}} - g_t^{\text{BestRootTemp}}}, 1\right\}^{g_t^{\text{RootHeatPower}}} \tag{S9}$$

$$v_d^{\text{RootCold}} = \min\left\{\frac{\min\{v_d^{\text{ARootTemp}} - g_t^{\text{BestRootTemp}}, 0\}}{g_t^{\text{MinRootGrowthTemp}} - g_t^{\text{BestRootTemp}}}, 1\right\}^{g_t^{\text{RootColdPower}}} \tag{S10}$$

$$v_d^{\text{AirStress}} = 1 - (1 - v_d^{\text{AirHeat}}) \cdot (1 - v_d^{\text{AirCold}}) \tag{S11}$$

$$v_d^{\text{RootStress}} = 1 - (1 - v_d^{\text{RootHeat}}) \cdot (1 - v_d^{\text{RootCold}}) \tag{S12}$$

$$v_d^{\text{AccuAirStress}} = \max\left\{v_{d-1}^{\text{AccuAirStress}} - \frac{v_{d-1}^{\text{AccuAirStress}} * v_{d-1}^{\text{MShootWeight}}}{g^{\text{AccuAirStressRecovery}}} + v_d^{\text{AirStress}}, 0\right\} \tag{S13}$$

$$v_d^{\text{AccuRootStress}} = \max\left\{v_{d-1}^{\text{AccuRootStress}} - \frac{v_{d-1}^{\text{AccuRootStress}} * v_{d-1}^{\text{MRootWeight}}}{g^{\text{AccuRootStressRecovery}}} + v_d^{\text{RootStress}}, 0\right\} \tag{S14}$$

$$v_d^{\text{AccuAirStressIndex}} = 1 - \frac{v_d^{\text{AccuAirStress}}}{v_d^{\text{AccuAirStress}} + g^{\text{AccuAirStress}}} \tag{S15}$$

$$v_d^{\text{AccuRootStressIndex}} = 1 - \frac{v_d^{\text{AccuRootStress}}}{v_d^{\text{AccuRootStress}} + g^{\text{AccuRootStress}}} \tag{S16}$$

$$v^{\text{WaterDensityIndex}} = e^{(-g^{\text{WaterPlantDensity}} * M^{Density} + g^{\text{WaterPlantDensity}})} \tag{S17}$$

$$v^{\text{LightDensityIndex}} = e^{(-g^{\text{LightPlantDensity}} * M^{Density} + g^{\text{LightPlantDensity}})} \tag{S18}$$

Equations (S7)-(S10) calculate heat and cold stresses based on air temperature and root temperature. Equation (S11) gives the overall stress for leaf, stem, and grain, and Equation (S12) gives the overall stress for root. Equation (S13) and (S14) calculate the accumulated air and root stress and the effects of accumulated stress are illustrated in the Equation (S15) and Equation (S16). Equations (S17) and (S18) determine the water and solar radiation absorption coefficients.

Water Module

$$v_d^{\text{MXylemCap}} = g_t^{\text{Xylem/Leaf}} \cdot v_{d-1}^{\text{MLeafWeight}} + g_t^{\text{Xylem/Stem}} \cdot v_{d-1}^{\text{MStemWeight}} \tag{S19}$$

$$v_d^{\text{TXylemCap}} = g_t^{\text{Xylem/Leaf}} \cdot v_{d-1}^{\text{TLeafWeight}} + g_t^{\text{Xylem/Stem}} \cdot v_{d-1}^{\text{TStemWeight}} \tag{S20}$$

$$\begin{aligned} v_d^{\text{RootWaterCap}} &= (1 - v_d^{\text{RootStress}}) \cdot \frac{g_t^{\text{RootWaterCap}}}{M^{\text{Density}}} \cdot v_d^{\text{AccuRootStressIndex}} \cdot v^{\text{WaterDensityIndex}} \\ &\cdot \sum_l [(v_{d,l}^{\text{MRootLayerLength}} + v_{d,l}^{\text{RootTLayerLength}}) \cdot E_{d,l}^{\text{SoilMoisture}}] \end{aligned} \tag{S21}$$

$$v_d^{\text{MWaterUptakeCap}} = \max\{v_d^{\text{MXylemCap}} - (v_{d-1}^{\text{MWater}} - v_{d-1}^{\text{MTranspiration}} - v_{d-1}^{\text{MWaterPhoto}}), 0\} \tag{S22}$$

$$v_d^{\text{TWaterUptakeCap}} = \max\{v_d^{\text{TXylemCap}} - (v_{d-1}^{\text{TWater}} - v_{d-1}^{\text{TTranspiration}} - v_{d-1}^{\text{TWaterPhoto}}), 0\} \tag{S23}$$

$$v_d^{\text{TotalWaterUptake}} = \min\left\{v_d^{\text{MWaterUptakeCap}} + v_d^{\text{TWaterUptakeCap}}, v_d^{\text{RootWaterCap}}\right\} \tag{S24}$$

$$v_d^{\text{MWaterUptake}} = \min\left\{v_d^{\text{MWaterUptakeCap}}, v_d^{\text{TotalWaterUptake}}\right\} \tag{S25}$$

$$v_d^{\text{TWaterUptake}} = v_d^{\text{TotalWaterUptake}} - v_d^{\text{MWaterUptake}} \tag{S26}$$

$$\begin{aligned} v_d^{\text{MWater}} &= \min\{v_d^{\text{MXylemCap}}, v_{d-1}^{\text{MWater}} - v_{d-1}^{\text{MTranspiration}} - v_{d-1}^{\text{MWaterPhoto}} \\ &+ v_d^{\text{MWaterUptake}}\} \end{aligned} \tag{S27}$$

$$\begin{aligned} v_d^{\text{TWater}} &= \min\{v_d^{\text{TXylemCap}}, v_{d-1}^{\text{TWater}} - v_{d-1}^{\text{TTranspiration}} - v_{d-1}^{\text{TWaterPhoto}} \\ &+ v_d^{\text{TWaterUptake}}\} \end{aligned} \tag{S28}$$

Equations (S19) and (S20) calculate the water storage and transport capacities in the xylem of the main crop and tiller. Equation (S21) determines capacity of water uptake at all soil layers, adjusted by stand count. Equations (S22) and (S23) calculate the maximal amount of water uptake by the main crop and tiller. Equation (S24) determines total water uptake by main crop and tiller, considering xylem capacity and root water availability. Equations (S25) and (S26) calculate actual water uptake by the main crop and tiller, with the main crop having priority over tiller to access water. Equations (S27) and (S28) update crop water storage balance in the main crop and tiller after transpiration and uptake.

Transpiration Module

$$v_d^{\text{TempTranspiration}} = 1 - e^{\min\{32 - E_d^{\text{AirTemp}}, 0\} \cdot g^{\text{TempTranspiration}}} \tag{S29}$$

$$v_d^{\text{HumidityTranspiration}} = e^{-E_d^{\text{Humidity}} \cdot g^{\text{HumidityTranspiration}}} \tag{S30}$$

$$v_d^{\text{EvapTranspiration}} = 1 - g^{\text{EvapTranspiration0}} \cdot e^{-E_d^{\text{Evap}} \cdot g^{\text{EvapTranspiration1}}} \tag{S31}$$

$$v_d^{\text{WindTranspiration}} = 1 - g^{\text{WindTranspiration0}} \cdot e^{-E_d^{\text{Wind}} \cdot g^{\text{WindTranspiration1}}} \tag{S32}$$

$$\begin{aligned} v_d^{\text{MTranspiration}} &= v_d^{\text{MWater}} \cdot v_d^{\text{TempTranspiration}} \cdot v_d^{\text{HumidityTranspiration}} \\ &\cdot v_d^{\text{EvapTranspiration}} \cdot v_d^{\text{WindTranspiration}} \end{aligned} \tag{S33}$$

$$\begin{aligned} v_d^{\text{TTranspiration}} &= v_d^{\text{TWater}} \cdot v_d^{\text{TempTranspiration}} \cdot v_d^{\text{HumidityTranspiration}} \\ &\cdot v_d^{\text{EvapTranspiration}} \cdot v_d^{\text{WindTranspiration}} \end{aligned} \tag{S34}$$

Equations (S29), (S30), (S31), and (S32) calculate the effects of air temperature, humidity, evaporation, and wind to transpiration, respectively. Equations (S33) and (S34) determine the amounts of transpiration for the main crop and tiller based on the previous four effects.

Biomass Module

$$v_d^{\text{PhotoLightCap}} = g_t^{\text{Photo/Light}} \cdot E_d^{\text{Light}} / M^{\text{Density}} \cdot v_d^{\text{AccuAirStressIndex}} \cdot v^{\text{LightDensityIndex}} \tag{S35}$$

$$v_d^{\text{MPhotoWaterCap}} = g_t^{\text{Photo/Water}} \cdot \min\{v_d^{\text{MWater}} - v_d^{\text{MTranspiration}}, g_t^{\text{PhotoWater/Transpiration}} \cdot v_d^{\text{MTranspiration}}\} \tag{S36}$$

$$v_d^{\text{TPhotoWaterCap}} = g_t^{\text{Photo/Water}} \cdot \min\{v_d^{\text{TWater}} - v_d^{\text{TTranspiration}}, g_t^{\text{PhotoWater/Transpiration}} \cdot v_d^{\text{TTranspiration}}\} \tag{S37}$$

$$v_d^{\text{MPhotoLeafCap}} = g_t^{\text{Photo/Leaf}} \cdot (1 - v^{\text{AirStress}}) \cdot v_{d-1}^{\text{MLeafWeight}} \cdot v_d^{\text{AccuAirStressIndex}} \tag{S38}$$

$$v_d^{\text{TPhotoLeafCap}} = g_t^{\text{Photo/Leaf}} \cdot (1 - v^{\text{AirStress}}) \cdot v_{d-1}^{\text{TLeafWeight}} \cdot v_d^{\text{AccuAirStressIndex}} \tag{S39}$$

$$v_d^{\text{MPhloemCap}} = g_t^{\text{Phloem/Leaf}} \cdot v_{d-1}^{\text{MLeafWeight}} + g_t^{\text{Phloem/Stem}} \cdot v_{d-1}^{\text{MStemWeight}} \tag{S40}$$

$$v_d^{\text{TPhloemCap}} = g_t^{\text{Phloem/Leaf}} \cdot v_{d-1}^{\text{TLeafWeight}} + g_t^{\text{Phloem/Stem}} \cdot v_{d-1}^{\text{TStemWeight}} \tag{S41}$$

$$v_d^{\text{MPhoto}} = \min\left\{v_d^{\text{PhotoLightCap}}, v_d^{\text{MPhotoWaterCap}}, v_d^{\text{MPhotoLeafCap}}, v_d^{\text{MPhloemCap}} - v_{d-1}^{\text{MBiomass}}\right\} \tag{S42}$$

$$v_d^{\text{TPhoto}} = \min\left\{v_d^{\text{PhotoLightCap}} - v_d^{\text{MPhoto}}, v_d^{\text{TPhotoWaterCap}}, v_d^{\text{TPhotoLeafCap}}, v_d^{\text{TPhloemCap}} - v_{d-1}^{\text{TBiomass}}\right\} \tag{S43}$$

$$v_d^{\text{MLeafGrowthCap}} = (1 - v_d^{\text{AirStress}}) \cdot \min\{g_t^{\Delta\text{Leaf0}} + g_t^{\Delta\text{Leaf1}} \cdot v_d^{\text{MLeafWeight}} + v_d^{\text{MLeafMS}}, g_t^{\text{Phloem/Leaf}} \cdot (v_{d-1}^{\text{MLeafWeight}} + v_{d-1}^{\text{TLeafWeight}})\} \tag{S44}$$

$$v_d^{\text{MRootGrowthCap}} = (1 - v_d^{\text{RootStress}}) \cdot (g_t^{\Delta\text{Root0}} + g_t^{\Delta\text{Root1}} \cdot v_d^{\text{MRootWeight}} + v_d^{\text{MRootMS}}) \tag{S45}$$

$$v_d^{\text{MStemGrowthCap}} = (1 - v_d^{\text{AirStress}}) \cdot \min\{g_t^{\Delta\text{Stem0}} + g_t^{\Delta\text{Stem1}} \cdot v_d^{\text{MStemWeight}} + v_d^{\text{MStemMS}}, g_t^{\text{Phloem/Stem}} \cdot (v_{d-1}^{\text{MStemWeight}} + v_{d-1}^{\text{TStemWeight}})\} \tag{S46}$$

$$v_d^{\text{MGrainGrowthCap}} = (1 - v_d^{\text{AirStress}}) \cdot (g_t^{\Delta\text{Grain0}} + g_t^{\Delta\text{Grain1}} \cdot v_d^{\text{MGrainWeight}} + v_d^{\text{MGrainMS}}) \tag{S47}$$

$$v_d^{\text{MGrowthCap}} = v_d^{\text{MLeafGrowthCap}} + v_d^{\text{MStemGrowthCap}} + v_d^{\text{MGrainGrowthCap}} + v_d^{\text{MRootGrowthCap}} \tag{S48}$$

$$v_d^{\text{TLeafGrowthCap}} = (1 - v_d^{\text{AirStress}}) \cdot \min\{g_t^{\Delta\text{Leaf0}} + g_t^{\Delta\text{Leaf1}} \cdot v_d^{\text{TLeafWeight}} + v_d^{\text{TLeafMS}}, g_t^{\text{Phloem/Leaf}} \cdot (v_{d-1}^{\text{MLeafWeight}} + v_{d-1}^{\text{TLeafWeight}})\} \tag{S49}$$

$$v_d^{\text{TRootGrowthCap}} = (1 - v_d^{\text{RootStress}}) \cdot (g_t^{\Delta\text{Root0}} + g_t^{\Delta\text{Root1}} \cdot v_d^{\text{TRootWeight}} + v_d^{\text{TRootMS}}) \tag{S50}$$

(S51)

$$v_d^{\text{TStemGrowthCap}} = (1 - v_d^{\text{AirStress}}) \cdot \min\{g_t^{\Delta\text{Stem0}} + g_t^{\Delta\text{Stem1}} \cdot v_d^{\text{TStemWeight}} + v_d^{\text{TStemMS}}, g_t^{\text{Phloem/Stem}} \cdot (v_{d-1}^{\text{MStemWeight}} + v_{d-1}^{\text{TStemWeight}})\} \quad \text{(S52)}$$

$$v_d^{\text{TGrainGrowthCap}} = (1 - v_d^{\text{AirStress}}) \cdot (g_t^{\Delta\text{Grain0}} + g_t^{\Delta\text{Grain1}} \cdot v_d^{\text{TGrainWeight}} + v_d^{\text{TGrainMS}}) \quad \text{(S53)}$$

$$v_d^{\text{TGrowthCap}} = v_d^{\text{TLeafGrowthCap}} + v_d^{\text{TStemGrowthCap}} + v_d^{\text{TGrainGrowthCap}} + v_d^{\text{TRootGrowthCap}} \quad \text{(S54)}$$

$$v_d^{\text{MGrowth}} = \min\{v_{d-1}^{\text{MBiomass}} + v_d^{\text{MPhoto}}, v_d^{\text{MGrowthCap}}\} \quad \text{(S55)}$$

$$v_d^{\text{TGrowth}} = \min\{v_{d-1}^{\text{TBiomass}} + v_d^{\text{TPhoto}} + v_{d-1}^{\text{MBiomass}} + v_d^{\text{MPhoto}} - v_d^{\text{MGrowth}}, v_d^{\text{TGrowthCap}}\} \quad \text{(S56)}$$

$$v_d^{\text{MBiomass}} = v_{d-1}^{\text{MBiomass}} + v_d^{\text{MPhoto}} - v_d^{\text{MGrowth}} - \max\{v_d^{\text{TGrowth}} - v_{d-1}^{\text{TBiomass}} - v_d^{\text{TPhoto}}, 0\} \quad \text{(S57)}$$

$$v_d^{\text{TBiomass}} = \max\{v_{d-1}^{\text{TBiomass}} + v_d^{\text{TPhoto}} - v_d^{\text{TGrowth}}, 0\} \quad \text{(S58)}$$

$$v_d^{\text{MWaterPhoto}} = v_d^{\text{MPhoto}} / g_t^{\text{Photo/Water}} \quad \text{(S59)}$$

$$v_d^{\text{TWaterPhoto}} = v_d^{\text{TPhoto}} / g_t^{\text{Photo/Water}} \quad \text{(S60)}$$

Equations (S35)-(S41) calculate the capacities of biomass accumulation due to solar radiation, crop water, leaf, and phloem capacity constraints, with radiation being adjusted by stand count. Equations (S42) and (S43) determine the actual biomass accumulation as the smallest of the four capacities; main crop has priority over tiller to use solar radiation. Equations (S44) to (S54) determine maximal growth for all organs of the main crop and tiller. Equations (S55) and (S56) give actual growths for the main crop and tiller. Equations (S57) and (S58) update the crop biomass reserve balance for the main crop and tiller. Equations (S59) and (S60) calculate water consumption for biomass production by the main crop and tiller.

Maintenance and Senescence Module

$$v_d^{\text{MLeafMS}} = \min\{g_t^{\text{LeafMS0}} + g_t^{\text{LeafMS1}} \cdot v_{d-1}^{\text{AirStress}}, 1\} \cdot v_{d-1}^{\text{MLeafWeight}} \quad \text{(S61)}$$

$$v_d^{\text{MRootMS}} = \min\{g_t^{\text{RootMS0}} + g_t^{\text{RootMS1}} \cdot v_{d-1}^{\text{RootStress}}, 1\} \cdot v_{d-1}^{\text{MRootWeight}} \quad \text{(S62)}$$

$$v_d^{\text{MStemMS}} = \min\{g_t^{\text{StemMS0}} + g_t^{\text{StemMS1}} \cdot v_{d-1}^{\text{AirStress}}, 1\} \cdot v_{d-1}^{\text{MStemWeight}} \quad \text{(S63)}$$

$$v_d^{\text{MGrainMS}} = \min\{g_t^{\text{GrainMS0}} + g_t^{\text{GrainMS1}} \cdot v_{d-1}^{\text{AirStress}}, 1\} \cdot v_{d-1}^{\text{MGrainWeight}} \quad \text{(S64)}$$

$$v_d^{\text{TLeafMS}} = \min\{g_t^{\text{LeafMS0}} + g_t^{\text{LeafMS1}} \cdot v_{d-1}^{\text{AirStress}}, 1\} \cdot v_{d-1}^{\text{TLeafWeight}} \quad \text{(S65)}$$

$$v_d^{\text{TRootMS}} = \min\{g_t^{\text{RootMS0}} + g_t^{\text{RootMS1}} \cdot v_{d-1}^{\text{RootStress}}, 1\} \cdot v_{d-1}^{\text{TRootWeight}} \quad \text{(S66)}$$

$$v_d^{\text{TStemMS}} = \min\{g_t^{\text{StemMS0}} + g_t^{\text{StemMS1}} \cdot v_{d-1}^{\text{AirStress}}, 1\} \cdot v_{d-1}^{\text{TStemWeight}} \quad \text{(S67)}$$

$$v_d^{\text{TGrainMS}} = \min\{g_t^{\text{GrainMS0}} + g_t^{\text{GrainMS1}} \cdot v_{d-1}^{\text{AirStress}}, 1\} \cdot v_{d-1}^{\text{TGrainWeight}} \quad \text{(S68)}$$

Equations (S61)-(S68) calculate the amount of photosynthate consumption for maintenance and senescence for leaf, root, stem, and grain, determined by organ weight and stress.

Growth Module

$$v_d^{\text{MLeafWeight}} = v_{d-1}^{\text{MLeafWeight}} - v_d^{\text{MLeafMS}} + v_d^{\text{MGrowth}} \cdot v_d^{\text{MLeafGrowthCap}} / v_d^{\text{MGrowthCap}} \tag{S69}$$

$$v_d^{\text{MRootWeight}} = v_{d-1}^{\text{MRootWeight}} - v_d^{\text{MRootMS}} + v_d^{\text{MGrowth}} \cdot v_d^{\text{MRootGrowthCap}} / v_d^{\text{MGrowthCap}} \tag{S70}$$

$$v_d^{\text{MRootLength}} = \max\{v_d^{\text{MRootWeight}} / g_t^{\text{RootDensity}}, v_{d-1}^{\text{MRootLength}}\} \tag{S71}$$

$$v_{d,l}^{\text{MRootLayerLength}} = \min\left\{ v_d^{\text{MRootLength}} - \sum_{k<l} v_{d,k}^{\text{MRootLayerLength}}, E_l^{\text{SoilLayer}} \right\} \tag{S72}$$

$$v_d^{\text{MStemWeight}} = v_{d-1}^{\text{MStemWeight}} - v_d^{\text{MStemMS}} + v_d^{\text{MGrowth}} \cdot v_d^{\text{MStemGrowthCap}} / v_d^{\text{MGrowthCap}} \tag{S73}$$

$$v_d^{\text{MStemHeight}} = \max\{v_d^{\text{MStemWeight}} / g_t^{\text{StemDensity}}, v_{d-1}^{\text{MStemHeight}}\} \tag{S74}$$

$$v_d^{\text{MGrainWeight}} = v_{d-1}^{\text{MGrainWeight}} - v_d^{\text{MGrainMS}} + v_d^{\text{MGrowth}} \cdot v_d^{\text{MGrainGrowthCap}} / v_d^{\text{MGrowthCap}} \tag{S75}$$

$$v_d^{\text{MShootWeight}} = v_d^{\text{MLeafWeight}} + v_d^{\text{MStemWeight}} + v_d^{\text{MGrainWeight}} \tag{S76}$$

$$v_d^{\text{TLeafWeight}} = v_{d-1}^{\text{TLeafWeight}} - v_d^{\text{TLeafMS}} + v_d^{\text{TGrowth}} \cdot v_d^{\text{TLeafGrowthCap}} / v_d^{\text{TGrowthCap}} \tag{S77}$$

$$v_d^{\text{TRootWeight}} = v_{d-1}^{\text{TRootWeight}} - v_d^{\text{TRootMS}} + v_d^{\text{TGrowth}} \cdot v_d^{\text{TRootGrowthCap}} / v_d^{\text{TGrowthCap}} \tag{S78}$$

$$v_d^{\text{TRootLength}} = \max\{v_d^{\text{TRootWeight}} / g_t^{\text{RootDensity}}, v_{d-1}^{\text{TRootLength}}\} \tag{S79}$$

$$v_{d,l}^{\text{TRootLayerLength}} = \min\left\{ v_d^{\text{TRootLength}} - \sum_{k<l} v_{d,k}^{\text{TRootLayerLength}}, E_l^{\text{SoilLayer}} \right\} \tag{S80}$$

$$v_d^{\text{TStemWeight}} = v_{d-1}^{\text{TStemWeight}} - v_d^{\text{TStemMS}} + v_d^{\text{TGrowth}} \cdot v_d^{\text{TStemGrowthCap}} / v_d^{\text{TGrowthCap}} \tag{S81}$$

$$v_d^{\text{TStemHeight}} = \max\{v_d^{\text{TStemWeight}} / g_t^{\text{StemDensity}}, v_{d-1}^{\text{TStemHeight}}\} \tag{S82}$$

$$v_d^{\text{TGrainWeight}} = v_{d-1}^{\text{TGrainWeight}} - v_d^{\text{TGrainMS}} + v_d^{\text{TGrowth}} \cdot v_d^{\text{TGrainGrowthCap}} / v_d^{\text{TGrowthCap}} \tag{S83}$$

$$v_d^{\text{TShootWeight}} = v_d^{\text{TLeafWeight}} + v_d^{\text{TStemWeight}} + v_d^{\text{TGrainWeight}} \tag{S84}$$

Equations (S69)-(S84) update leaf weights, root weights, root lengths, stem weights, stem heights, grain weights, main crop weights, and tiller weights, after considering maintenance and growth respiration. Root lengths and stem heights are assumed to be non-decreasing; when maintenance exceeds growth, the weight reduces, but length and height stay the same, which reduces density below the regular value. When growth biomass is replenished, it restores the density to the regular value first before it grows the length and height.

2 COMPARING TRAINED GENOTYPE VARIABLES

Figure S1 displays the probability density curves of 56 parameters across three training scenarios: 1) the 2021 dataset (red), 2) the 2022 dataset (blue), and 3) the combined dataset (green). Note that all subplots are kernel density estimate (KDE) plots. While KDE plots represent a probability density function, the y-axis values can exceed 1 because probability density is not the same as probability. Instead of focusing on the y-axis values, attention should be given to the shape of the curve, keeping in mind that the area under the KDE curve always sums to 1. Some variables (e.g., $g^{\text{BestAirTemp}}$, $g^{\text{MinRootTemp}}$, g^{Root0}, g^{RootMS0})

exhibit similar results across all three datasets. Some variables (e.g., $g^{\text{Xylem/Stem}}$, $g^{\text{RootStressRecover}}$) show noticeable differences in performance depending on the dataset used for training.

The similar trained variables comparison results indicate that some variable like $g^{\text{BestAirTemp}}$, $g^{\text{MinRootTemp}}$, and $g^{\text{MaxRootTemp}}$ are robust and less sensitive to the choice of training dataset. hese temperature thresholds for sorghum growth do not vary significantly with differences in weather conditions, suggesting that the proposed model can effectively separate the influence of genotype and environment. In contrast, the comparison results for variables such as $g^{\text{Xylem/Stem}}$ and $g^{\text{RootStressRecover}}$ suggest that the model tends to favor part of the data when there are discrepancies between the 2021 and 2022 observed records. This bias could be mitigated by incorporating additional training datasets to improve the model's generalization ability.

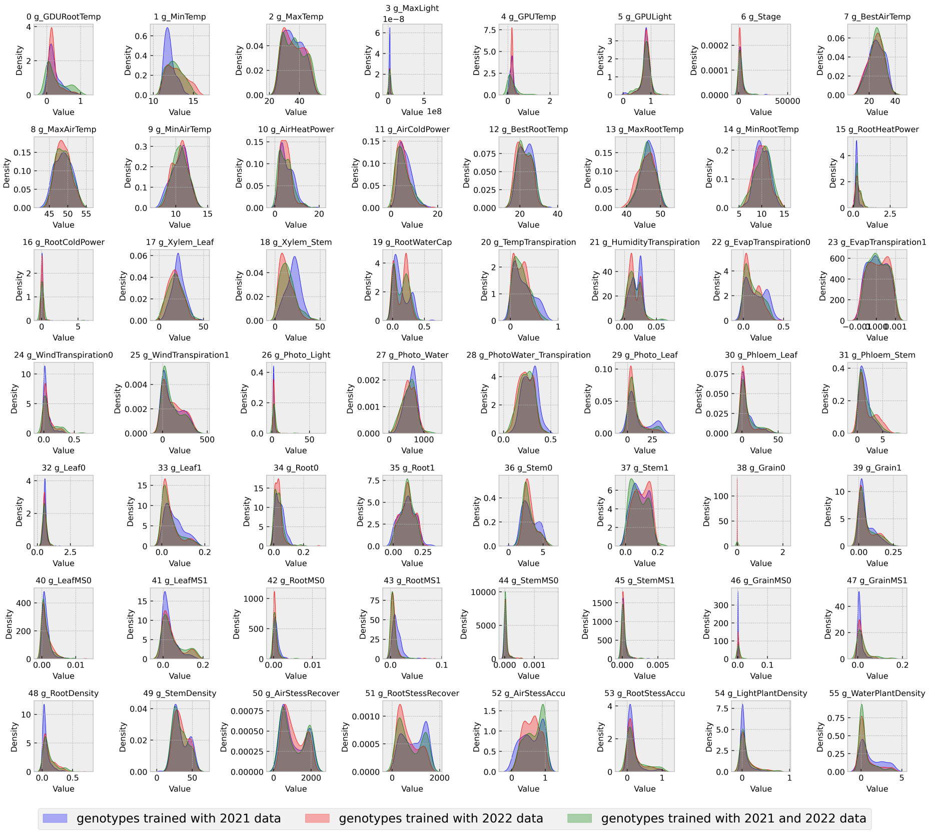

Figure S1. Trained Genotypes Variables Comparison
